# Multi-modal clustering reveals event-free patient subgroup in colorectal cancer survival

**DOI:** 10.1038/s41540-025-00557-3

**Published:** 2025-08-02

**Authors:** Nikita Janakarajan, Guillaume Larghero, María Rodríguez Martínez

**Affiliations:** 1 IBM Research Europe, Rüschlikon, Switzerland; 2 D-INFK, ETH Zürich, Zürich, Switzerland; 3 D-BSSE, ETH Zürich, Zürich, Switzerland

**Keywords:** Cancer, Computational biology and bioinformatics

## Abstract

Colorectal cancer (CRC) benefits from a multi-omics-based stratification in the context of survival. Our TCGA-based study employs targeted feature selection and unsupervised clustering to stratify patients based on disease-specific survival, identifying an event-free subgroup undetectable with unimodal data or established consensus molecular subtypes. An analysis of variance and gene set enrichment coupled with clinical characterisation of the clusters reveal findings that support multi-omics-driven precision medicine in CRC.

## Introduction

Multi-omics data combines quantifying information of various biomolecules and molecular processes that constitute a cell or tissue. By considering these different, interacting processes, multi-omics data exhibit the potential to unravel additional insights into phenomena of interest over using only single-omics data. One such phenomenon that can particularly benefit from a multi-omics based analysis is cancer, an area that is not well understood and where such data could offer meaningful insights.

Colorectal cancer (CRC), the third most common cancer in the world, is responsible for ≈ 9% of all cancer-related deaths worldwide in 2022^1^. To reduce the mortality rate, it is imperative to understand its molecular basis to identify effective treatment strategies. A major study carried out by the Consortium for Colorectal Cancer Subtyping utilised gene expression data to identify 4 consensus molecular subtypes (CMS) of CRC^2^. While the study resulted in a high-level characterisation of CRC subtypes, extending it to multi-omics only produced partially similar clusters^3^. The multi-omics-derived clusters outperformed CMS clusters in predicting patient survival, which showed no prognostic value for the cohorts in their study (TCGA COADREAD and CCLE)^3^. This prompted us to conduct a multi-omics analysis of CRC in the context of the rarely-studied disease-specific survival (DSS).

**Fig. 1:**
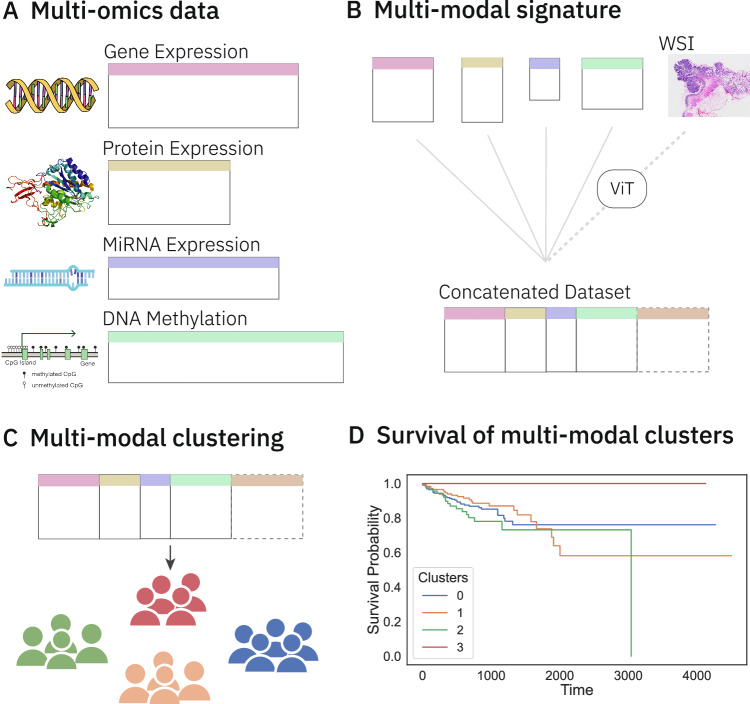
Overview of the workflow. **A** Multi-omics data pertaining to colorectal cancer (CRC) is collected from TCGA. **B** Signatures for each omics type are retrieved from the literature. These signatures are used to subset each omics dataset, which are then concatenated to form a multi-omics dataset. Whole slide image (WSI) embeddings from a Vision Transformer (ViT) are concatenated to this multi-omics dataset to form a multi-modal dataset. **C** Unsupervised clustering on these datasets groups patients into distinct clusters. **D** The clusters are analysed for unique survival patterns through survival analysis methods such as Kaplan-Meier curves and significance testing by Peto-weighted pairwise log-rank test. Icons from Wikimedia Commons: DNA helix by Leyo (public domain); miRNA by DBCLS (CC BY 4.0); protein by Emw (CC BY-SA 3.0); WSI by Ed Uthman, MD (CC BY-SA 2.0); DNA methylation by Mariuswalter (CC BY-SA 4.0).

However, handling multi-omics data comes with its challenges. It has been shown that using multi-omics data in its entirety does not have much benefit for survival prediction over using clinical or gene expression data, either due to interference from noise or high dimensionality of the data^4,5^. We exploit signatures identified in prior studies from each omics data to tackle these challenges. Our main objective is to assess the potential of relying on multi-omics data to obtain relevant disease characterisation insights. In this context, relying solely on unsupervised approaches guarantees limiting biases arising from taking a task-specific perspective to the problem. To this end, we perform unsupervised clustering over these multi-omics signatures and identify a 0-event group with significantly different survival behaviour (Fig. 1). In addition to clinical characterisation of the obtained groups, an in-depth analysis of this significantly different survival group through analysis of variance (ANOVA) and gene set enrichment analysis (GSEA) highlights the important contributing features and the associated pathways, providing additional insights into the clusters generated by multi-omics data. We extend multi-omics data to include whole slide images and observe similar results.

Our goal is to show that multi-omics data have benefits in stratifying patients to obtain relevant disease characterisation insights over single omics. To this end, we work directly with known CRC signatures from each omics data and evaluate this on DSS. Despite using DSS as a criterion for evaluation, we show how an unbiased and unsupervised approach to multi-modal data analysis naturally leads to insights not apparent when using single modalities.

Selecting highly relevant features as opposed to compressing all features into a lower-dimensional space has its advantages. First, any inferences made on account of these features are not dependent on the quality of transformations, as in the case of compression. Second, retaining the original values makes interpreting the results easier. Third, the reduction to a smaller, relevant subset minimises the risk of introducing spurious relationships. And four, the impact of irrelevant and redundant features on downstream tasks is reduced. For gene expression, we use 40 genes identified by ref. ^6^ as being significantly associated with CRC and the defined CMS. For DNA methylation, we select all the probes mapping to differentially methylated genes associated with CRC prognosis as found in refs. ^7,8^, in addition to 16 CpG sites identified by ref. ^9^, 26 markers that can distinguish between CIMP-Negative and CIMP-Low tumours by ref. ^10^ and 5 markers that are differentially expressed compared to normal adjacent tissue by ref. ^11^, amounting to a total of 82 probes in common with the TCGA COADREAD dataset. A collection of miRNA signatures found to be significantly associated with CRC progression and metastasis^12–14^, including a miRNA signature that can discriminate early stage CRC^15–17^, and a novel tumour suppressor miRNA^18^, make up the 30 miRNAs chosen for the study. Lastly, two major proteomics studies^19,20^ and a study relating proteomics to prognosis^21^ led to the finding of 11 proteins differentially expressed in CRC for our study.

To highlight the benefit of combining different omics data in patient stratification, we use CMS^2^ as the baseline for our analyses. Grouping patients by CMS results in 4 different clusters, one for each subtype. In this experiment, we compare the distribution of DSS in baseline CMS clusters against clusters created by similarity in (i) a unimodal setting: a subset of gene signatures associated with CMS, (ii) a multi-omics setting: comprising (i) and select markers from other omics data such as DNA methylation, miRNA expression and protein expression, and (iii) a multi-modal setting: comprising (ii) and whole slide images. The optimal number of clusters for each dataset is determined using the elbow method, and is found to be *K* = 4 for all datasets, consistent with the CMS groupings. The results of the elbow method are shown in Supplementary Fig. S3, and Supplementary Table S2 summarises the average silhouette score for the number of clusters considered to verify the quality of the clusters. Silhouette score analyses and PCA visualisations for the chosen optimal number of clusters are illustrated in Supplementary Fig. S4. We additionally study the stability of the clusters generated by (ii) and (iii) by repeating the experiment five times, each time with a different seed. The results of this analysis are discussed in Supplementary Note S2.1. From the unimodal datasets, we select gene expression for comparison as it is the most widely used omics data. For reference, results of the other unimodal datasets, namely, DNA methylation, protein expression, miRNA expression, and whole slide images are discussed in Supplementary Note S3.

**Fig. 2:**
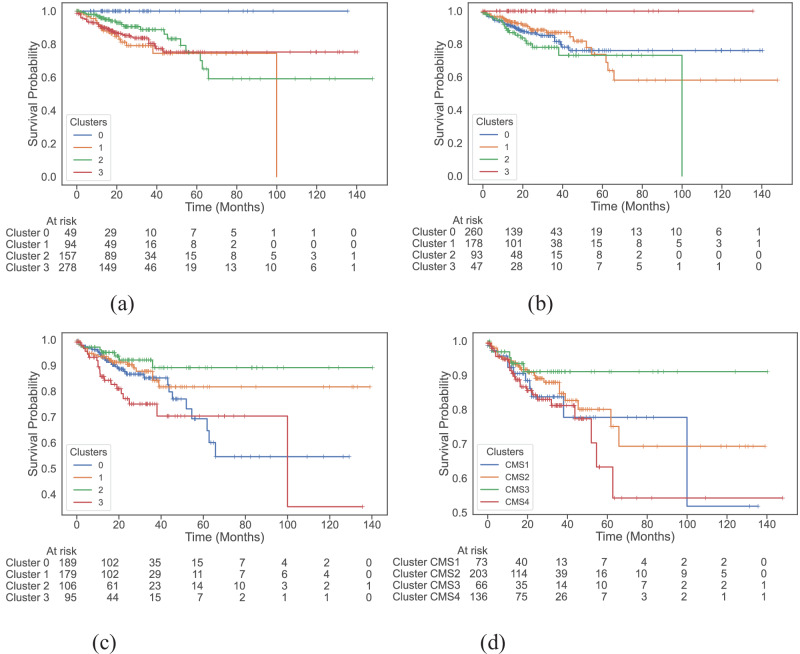
Cluster-survival association study. Kaplan-Meier plots of disease-specific survival (DSS) across clusters found by **a** multi-modal dataset, **b** multi-omics dataset, **c** colotype gene signatures, and **d** consensus molecular subtypes. The multi-modal and multi-omics datasets are able to identify an all-surviving cluster of patients with high significance.

We examine survival functions of the different clusters with the help of Kaplan-Meier plots (Fig. 2) and compute their pairwise significance using the Peto-weighted pairwise log-rank test (Table 1). We see that both multi-modal and multi-omics datasets identify a cluster of patients that experience no event at all (Fig. 2a, b, respectively). The all-surviving cluster 3 identified by multi-omics data has a significantly better survival rate than cluster 0 (*p* = 0.01), cluster 1 (*p* = 0.01), and cluster 2 (*p* < 0.005). Similarly, the multi-modal dataset isolates cluster 0 as the all-surviving group, and this cluster has a significantly better survival rate compared to cluster 1 (*p* < 0.005), cluster 2 (*p* = 0.01), and cluster 3 (*p* < 0.005). The addition of whole slide images to multi-omics improves the overall significance of differences between clusters by slightly altering cluster composition.

**Table 1:** Comparison of *p*-values computed using Peto-weighted log-rank statistics across different modalities to identify significantly different clusters in disease-specific survival

Multi-modal		
*cluster*_*i*_	*cluster*_*j*_	*p*–*value*
0	1	<**0.005**
0	2	**0.01**
0	3	<**0.005**
1	2	0.09
1	3	0.55
2	3	0.16

Multi-omics		
0	1	0.57
0	2	0.22
0	3	**0.01**
1	2	0.13
1	3	**0.01**
2	3	<**0.005**

Gene expression		
0	1	0.39
0	2	0.05
0	3	0.11
1	2	0.32
1	3	**0.02**
2	3	<**0.005**

Consensus Molecular Subtypes		
CMS1	CMS2	0.50
CMS1	CMS3	0.20
CMS1	CMS4	0.64
CMS2	CMS3	0.30
CMS2	CMS4	0.15
CMS3	CMS4	0.06

Significant *p*-values < 0.05 are highlighted in bold.

Further observations of these Kaplan-Meier curves (Fig. 2) show distinguishable survival trends between clusters with time. For instance, in the clusters generated by the multi-omics dataset (Fig. 2b), roughly after 15 months, the survival probability of patients in cluster 2 is lower than patients in other clusters, and the survival probability of patients beyond 10-years in this cluster is 0, suggesting worse 10-year survival compared to the rest. A similar trend is displayed by clusters from the multi-modal dataset, where patients in cluster 1 have a survival probability of 0 beyond 10 years.

We then look into the features contributing to the clusters by performing an ANOVA test between all cluster pairs. A binary heatmap of the top 10 discriminating features between cluster pairs for the multi-omics data (Supplementary Fig. S5) and multi-modal data (Supplementary Fig. S6) shows genes and DNA methylation probes as major contributors. For insights into the all-surviving cluster, we perform a gene set enrichment analysis against KEGG (2021)^22,23^ and MSigDB Hallmark (2020)^24,25^ gene sets. In the case of multi-omics, we select the union of all features that are significantly different (corrected *p*-value < 0.001) between clusters 0-3, 1-3, and 2-3, as cluster 3 is the all-surviving cluster. The discriminating features of cluster 3 are significantly enriched (adjusted *p*-value < 0.05) in four MSigDB Hallmark gene sets - (i) Unfolded Protein Response (adjusted *p*-value 0.0405), (ii) UV Response Downregulation (adjusted *p*-value 0.0405), (iii) Epithelial Mesenchymal Transition (EMT) (adjusted *p*-value 0.0418) and, (iv) G2-M Checkpoint (adjusted *p*-value 0.0418). The unfolded protein response signalling pathways can switch cell survival to cell death under endoplasmic reticulum stress, and their association with colorectal cancer is largely understudied^26^. Similarly, the role of genes downregulated due to UV radiation, such as receptor tyrosine kinases^27^ are also understudied, and findings from this multi-omics based clustering warrant a deeper investigation into the role of these two processes on colorectal cancer prognosis and progression. The EMT process has been associated with cancer progression^28^, and studies have investigated therapies against it^29^. This process could potentially be relatively downregulated in patients belonging to cluster 3. The G2-M checkpoint prevents cells from entering mitosis when DNA is damaged, and a stable G2 arrest helps to protect the genome and suppress tumourigenesis^30^. We repeat the same experiment with the multi-modal clusters and find the same results but with higher significance - (i) Unfolded Protein Response (adjusted *p*-value 0.0399), (ii) UV Response Downregulation (adjusted *p*-value 0.0399), (iii) Epithelial Mesenchymal Transition (EMT) (adjusted *p*-value 0.0411) and, (iv) G2-M Checkpoint (adjusted *p*-value 0.0411).

Given our findings, we perform a few sanity checks on our data to validate our results. First, we repeat the multi-omics clustering experiments with only patients who have all modalities recorded. This case also elicits an all-surviving patient group, thus ruling out any imputation artefacts (Supplementary Fig. S7). Additionally, we compare the top 10 discriminating features in this case to the ones obtained from imputed multi-omics data, and find significant overlap (Supplementary Note S2.3). Second, as the clustering results of multi-omics and multi-modal data are similar, we determine if there are any redundancies between them by computing the correlation between sample distances in the omics and image spaces (Supplementary Note S2.4) and find no correlations (pearsonr statistic = 0.093, *p* value=7.51e−127). Third, we perform a clinico-pathological characterisation of the clusters obtained from both multi-omics and multi-modal data (Supplementary Figs. S10–S20) to not only show that confounding factors such as age and gender have no effect on the clusters but also to highlight the utility of multi-omics signatures in stratifying key clinical variables (Supplementary Note S2.5). Fourth, we check for any information loss due to representing whole slide images by a simplified mean of all patches. To this end, we implement a bag-of-patches approach based on bag-of-visual-words^31–33^ using both normalised counts and term frequency-inverse document frequency metrics to create two versions of representations that capture local information. On repeating the multi-modal analysis using both representations, we find no differences in the clustering results aside from an increase in correlation of sample distances from 0.093 to approximately 0.17 between the omics and image spaces (Supplementary Note S2.6). Fifth, and finally, we validate our findings on a second dataset - CPTAC^34^. As there is a mismatch of available modalities between TCGA and CPTAC, we use a proxy to assign cluster labels to CPTAC patients. A Kaplan-Meier survival analysis with respect to overall-survival (OS) reveals similarities to TCGA-based multi-omics clusters - an all-surviving cluster (albeit with only 1 patient) and a cluster having survival probability 0 after approximately 45 months (Supplementary Fig. S25). This proxy study is discussed in Supplementary Note S2.7.

Comparing our findings to the baseline CMS (Fig. 2d), which are not significantly different from one another in terms of DSS (Table 1), we see an improvement. The prognostic power of CMS is limited to CMS4, which is associated with worse overall survival^2^. However, patients can also exhibit mixed CMS due to intra-tumour heterogeneity, which makes relying solely on the gene-expression-based CMS classes insufficient for prognosis^35^. To further demonstrate the benefits of using a multi-omics-based approach, we plot the distribution of CMS in the clusters identified by multi-omics and multi-modal data (Supplementary Fig. S15). The poor 10-year survival cluster has a majority of CMS4 patients followed by CMS1 patients, which is in line with previous findings^2,36^. However, we see that the all-surviving cluster is primarily a mix of CMS1 and CMS3 clusters with no CMS2 patients, despite it being associated with the best overall survival^2^. We believe multi-omics signatures are able to capture heterogeneity in the data, especially given the effect of the complex interplay between BRAF mutation, KRAS mutation, microsatellite instability (MSI), and CpG-island methylator phenotype (CIMP) on survival^37,38^. For instance, the separation of CMS1 patients into the poor and all-surviving clusters suggests that multi-omics signatures seem to take into account this complex interplay between high MSI and other factors. Moreover, by directly working with gene signatures instead of CMS, we already see significant differences in the survival rate between some clusters as seen in their Kaplan-Meier plot (Fig. 2c, Table 1). Thus, given the possibility of patients having heterogeneous CMS characterisations and with no way to currently address this, the multi-view provided by multi-omics data could help capture this intra-tumour heterogeneity, leading to better survival characterisation.

The benefit of our findings from multi-omics and multi-modal datasets lies in the potential advancement of personalised medicine research. The identification of a significantly different cluster unique to patients who experience no events with both multi-modal and multi-omics data (Table 1) suggests that the current treatment strategy is potentially working on this group of patients. Even if these patients were not subject to any treatment, it would mean that their general prognosis is positive, and further research in therapies for CRC should be directed towards other groups, particularly those clusters with a seemingly poor 10-year survival rate. This highlights the utility of incorporating information from multiple sources in patient stratification. Information from a single source could be biased, especially if it is not yet understood how the different elements that make up our complex system interact.

In conclusion, the inclusion of information from other complex biological systems in any analysis is often limited by the number of observations. But this does and should not prevent us from exploring their combined utility in getting one step closer to understanding biological phenomena. Although we take the classical approach of performing feature selection first by selecting markers that have been found to be associated with CRC in prior works, several methods exist that can perform feature selection automatically and learn non-linear relationships between the features that could be useful for patient stratification. Future studies could carefully explore these methods. Another avenue for further analysis is in the combination of signatures that one selects from different omics data. It is plausible that different combinations of features could produce better or worse stratification, and a study on this sensitivity to feature relevance would provide a reliable guideline to feature selection for patient stratification.

## Methods

### Data

We select the TCGA Colon Adenocarcinoma and Rectum Adenocarcinoma (COADREAD) dataset^39^ for our experiments. This publicly available dataset has the biggest collection of multi-omics data, including corresponding whole slide tissue images. Of the 7 data types available, we selected gene expression, protein expression, miRNA expression, DNA methylation values, and whole slide images for our study.

There are three types of survival values associated with this dataset - overall survival (OS), disease-specific survival (DSS), and progression-free interval (PFI). We select DSS for our analysis as it encodes events specifically caused by the disease instead of overall survival, which records all events. To this end, we consider only primary tumours for our analysis, as deaths of metastatic patients may be attributed to any one or more of the affected organs and/or tissues.

Additionally, we consider the CPTAC colon cancer dataset^34^ to validate our findings. Of the 3 modalities available, we selected gene expression and miRNA expression for our study. We do not consider proteomic expression as the technology used to measure it differs from the one used in TCGA COADREAD. There are 2 types of survival values associated with this dataset - overall survival (OS) and disease-free survival (DFS). We select OS for our validation experiment as it is closest to DSS in meaning.

#### Feature selection

Given that there are a large number of features compared to samples, it is imperative to reduce the dimensions of the omics datasets before proceeding with our analysis to draw reliable conclusions from them. We take the classical route of probing through literature for each data type and selecting omics features that have been proven by independent studies to be significantly associated with some characteristics of CRC. Keeping in mind the low sample size, we limit feature selection such that each data type contributes a similar number of features, thereby reducing selection bias. The intersection of the shortlisted features from literature and the available features in our dataset makes up the final subset used in our experiments. The list of features and their sources used for each data type is provided in Supplementary Note S1.

Whole slide images (WSI) are processed following the pipeline proposed in ref. ^40^. Most WSIs are scanned at 40x magnification (0.25 *μm*/pixel), while some others are scanned at 20x magnification (0.5 *μm*/pixel). Since the original images are high resolution, they are first split into patches and scaled down to 224 × 224 pixels. The pipeline uses Canny edge detection, as implemented by opencv, with lower threshold 40 and upper threshold 100 to identify edges in each patch. It is rejected if the patch has less than or equal to 2 edges. These patches are then Macenko normalised^41^. Once the patches are processed, they are passed into a pre-trained Vision Transformer (google/vit-base-patch16-224 from HuggingFace)^42^ to extract 768-dimensional embeddings. The choice of a non-histopathology fine-tuned model guarantees that the TCGA whole slide images were never a part of the training cohort and thus avoids issues with over-fitting and bias.

#### Data pre-processing

The selected subset of the gene expression dataset is first transformed from counts to log-FPKM (Fragments Per Kilobase per Million mapped fragments) normalised values. All omics datasets are then standardised such that each column has 0 mean and unit variance. To make the merging of image data with the omics data easier, the patches are aggregated to a single representation. We explore three methods of aggregation - mean, normalised count bag-of-patches, and TF-IDF bag-of-patches. The images are not processed any further.

##### WSI bag-of-patches

We implement a bag-of-visual-words approach^31–33^ to retrieve image representations. The images are processed into patches by the pipeline described in Section 1.1.1. Each patch is a 768-dimensional embedding and is independently normalised before fitting a K-means model as implemented by scikit-learn^43^ on it. The patches are normalised to ensure the cosine metric is used to compute distances. The number of clusters determines the representation size of the image. To this end, we consider k = [8,16,32,64,128] clusters and pick the optimal number of clusters k=32 using the elbow method following the Kneedle algorithm^44^. Each patch is assigned to a cluster. The patches are then grouped by patient ID and aggregated by count. This leaves us with a count matrix, where each row is a 32-dimensional vector for a given patient, each column represents a cluster ID, and the value is the number of patches of a given patient assigned to a given cluster. This matrix is then normalised to obtain a normalised count bag-of-patches representation and transformed to a Term Frequency-Inverse Document Frequency (TF-IDF) vector to obtain TF-IDF bag-of-patches.

#### Creating datasets

For unimodal experiments, the datasets are used as is. To create multi-omics and multi-modal (multi-omics with images) datasets, we perform an outer-join of the unimodal datasets, which causes the number of samples to exceed any given unimodal dataset. This is advantageous because we now have more samples to learn from, as the missing information from one data type is compensated for by information from another. As the outer-join results in some samples missing certain features, missing values are handled by a simple median imputation feature-wise. Supplementary Table S1 provides details on the number of missing values from each feature.

### Unsupervised clustering

We perform unsupervised clustering using the K-means++ algorithm^45^. K-means++ improves upon standard K-means^46^ by using a probability-based sampling method to select initial cluster centroids, which accelerates convergence. The first centroid is randomly selected from the dataset. Each subsequent centroid is chosen with probability proportional to the square of its distance from the nearest existing centroid. The scikit-learn^43^ method implements the greedy version of K-means++, running several trials at each sampling step and choosing the best centroid among them. This method then divides patients into K distinct clusters with the goal of minimising variance within a cluster. Thus, patients with a higher feature similarity or lower distances, as measured by the Euclidean metric, are assigned to the same cluster. By not providing the survival labels, we create sub-groups of patients only based on similarities in their features; hence, any implications the data may have on survival can be studied in post-clustering analyses.

#### Choosing the optimal number of clusters

The optimal number of clusters *K* for each dataset is determined using the elbow method complemented by the average silhouette score. For each value of *K*, the inertia or the within-cluster sum of squared errors is computed. The *K* at the bending point or ”elbow” of the inertia vs *K* plot represents the optimal *K* that balances the trade-off between minimising the inertia and over-fitting by having too many clusters. This elbow point is determined using the Kneedle Algorithm^44^ as implemented by the kneed Python package. Additionally, we compute the average silhouette scores for each cluster to verify the quality of the computed optimal number of clusters.

#### Evaluating cluster-survival association

The clusters of patients obtained from K-means on each dataset are evaluated for any associations with survival. The multi-omics representations of patients are reduced to cluster assignments, which are then mapped to the survival labels if available. In this analysis, no matter the dataset, we only look at patients for whom the survival information is available. Additionally, each cluster’s Kaplan-Meier curves^47^ are plotted to visualise survival curves. This is coupled with a Peto-weighted pairwise log-rank test between all clusters to compute significance in the differences between them. The Peto test is a variant of the log-rank test, which weights the i-th failure time by the survival estimate ~ *s*(*t*(*f*)) calculated over all groups combined, assigning more weight to earlier events^48^.

#### Analysis of variance (ANOVA)

The ANOVA test is performed between each pair of clusters to identify significantly discriminating features. The f_oneway function from the scipy.stats python package is used to compute the f-scores and *p*-values for each feature and each pair of clusters. The *p*-values are corrected by multiplying them with a correction factor, calculated as the product of the total number of cluster pairs and the total number of features considered. We get the f-score and *p*-value associated with each feature from this test. The higher the f-score, the lower the *p*-value, and the higher the feature importance in distinguishing between the 2 clusters.

#### Gene Set Enrichment Analysis (GSEA)

Gene set enrichment analysis is carried out using the gseapy Python package. The gene list comprises the union of all features identified by ANOVA that are significantly different (*p*-value < 0.001) and hence discriminatory for the cluster of interest (cluster 3 in the case of multi-omics). Since we use multi-omics signatures, molecules other than genes, i.e, proteins, DNA methylation probes, and miRNAs, are mapped to their corresponding genes. For proteins, we used GeneCards^49,50^. The DNA methylation probes are mapped using the probemap aligned with the hg19 reference genome that comes with the dataset on Xena Browser^51^. The miRNAs are mapped to their target genes using miRmap^52^. As the miRNAs in miRmap are defined by their MIMAT IDs (miRBase accession IDs), we use a miRNA ID converter tool provided with the miRandola database^53^ to convert the MIMAT IDs into current miRNA nomenclature (miRBase IDs). miRmap returns ensembl transcripts as targets which are then mapped to gene names using the mygene Python package, setting *scopes* to ”ensembl.transcripts”. The background genes for GSEA are all the features that make up our multi-omics dataset, mapped to gene names in the same way as described. We then use the enrichr method from gseapy to perform gene set enrichment analysis against MSigDB Hallmark (2020)^24,25^, and KEGG^22,23^ gene sets. This produces a list of enriched pathways, and the significant pathways (*p*-value < 0.05) are selected based on the adjusted *p*-value as computed by the GSEA tool.

## Supplementary information

Supplementary information

## Data Availability

The TCGA COADREAD and CPTAC Colon Cancer datasets are available for download on the Genomic Data Commons Portal (https://portal.gdc.cancer.gov). The datasets used in this study were downloaded from the Xena Browser (https://xenabrowser.net/datapages/). Data and results for reproducibility are available at https://zenodo.org/records/14604885.

## Code availability

The code for reproducibility is hosted at https://github.com/IBM/multimodal-survival-analysis.

## References

[1] Global cancer burden growing, amidst mounting need for services — who.int. https://www.who.int/news/item/01-02-2024-global-cancer-burden-growing--amidst-mounting-need-for-services. [Accessed 17-09-2024]. PMC11115397 38438207

[2] Guinney, J. et al. The consensus molecular subtypes of colorectal cancer. *Nat. Med.* **21**, 1350–1356 (2015). 26457759 10.1038/nm.3967 PMC4636487

[3] Ronen, J., Hayat, S. & Akalin, A. Evaluation of colorectal cancer subtypes and cell lines using deep learning. *Life Sci. Alliance* **2**, e201900517 (2019). 10.26508/lsa.201900517 PMC6892438 31792061

[4] Wissel, D., Rowson, D. & Boeva, V. Systematic comparison of multi-omics survival models reveals a widespread lack of noise resistance. *Cell Rep. Methods* **3**, 100461 (2023). 10.1016/j.crmeth.2023.100461 PMC10162996 37159669

[5] Herrmann, M., Probst, P., Hornung, R., Jurinovic, V. & Boulesteix, A.-L. Large-scale benchmark study of survival prediction methods using multi-omics data. *Brief. Bioinforma.* **22**, bbaa167 (2021). 10.1093/bib/bbaa167 PMC8138887 32823283

[6] Buechler, S. A. et al. Colotype: a forty-gene signature for consensus molecular subtyping of colorectal cancer tumors using whole-genome assay or targeted RNA-sequencing. *Sci. Rep.* **10**, 1–13 (2020). 32694712 10.1038/s41598-020-69083-y PMC7374173

[7] Hajebi Khaniki, S., Shokoohi, F., Esmaily, H. & Kerachian, M. A. Analyzing aberrant dna methylation in colorectal cancer uncovered intangible heterogeneity of gene effects in the survival time of patients. *Sci. Rep.* **13**, 22104 (2023). 38092774 10.1038/s41598-023-47377-1 PMC10719305

[8] Ma, Y. et al. Genome wide identification of novel DNA methylation driven prognostic markers in colorectal cancer. *Sci. Rep.* **14**, 15654 (2024). 38977698 10.1038/s41598-024-60351-9 PMC11231291

[9] Onwuka, J. U. et al. A panel of DNA methylation signatures from peripheral blood may predict colorectal cancer susceptibility. *BMC Cancer* **20**, 1–11 (2020). 10.1186/s12885-020-07194-5 PMC7382833 32711505

[10] van Den Berg, I. et al. A panel of DNA methylation markers for the classification of consensus molecular subtypes 2 and 3 in patients with colorectal cancer. *Mol. Oncol.* **15**, 3348–3362 (2021). 34510716 10.1002/1878-0261.13098 PMC8637568

[11] Baharudin, R. et al. Epigenome-wide dna methylation profiling in colorectal cancer and normal adjacent colon using Infinium Human Methylation 450k. *Diagnostics* **12**, 198 (2022). 35054365 10.3390/diagnostics12010198 PMC8775085

[12] Luo, X., Burwinkel, B., Tao, S. & Brenner, H. MicroRNA signatures: novel biomarker for colorectal cancer? *Cancer Epidemiol. Biomark. Prev.* **20**, 1272–1286 (2011). 10.1158/1055-9965.EPI-11-0035 21551242

[13] Huang, X. et al. Dissecting miRNA signature in colorectal cancer progression and metastasis. *Cancer Lett.* **501**, 66–82 (2021). 33385486 10.1016/j.canlet.2020.12.025

[14] Wang, X. et al. Identification of the miRNA signature and key genes in colorectal cancer lymph node metastasis. *Cancer Cell Int.* **21**, 1–12 (2021). 34315491 10.1186/s12935-021-02058-9 PMC8314594

[15] Gasparello, J. et al. A distinctive microRNA (miRNA) signature in the blood of colorectal cancer (CRC) patients at surgery. *Cancers* **12**, 2410 (2020). 32854257 10.3390/cancers12092410 PMC7564483

[16] Sheng, S. et al. Mir-144 inhibits growth and metastasis in colon cancer by down-regulating Smad4. *Biosci. Rep.* **39**, BSR20181895 (2019). 30745456 10.1042/BSR20181895 PMC6395301

[17] Cui, H. et al. Igf2-derived mir-483 mediated oncofunction by suppressing dlc-1 and associated with colorectal cancer. *Oncotarget* **7**, 48456 (2016). 27366946 10.18632/oncotarget.10309 PMC5217031

[18] Liang, J. et al. Epigenetically regulated mir-1247 functions as a novel tumour suppressor via mycbp2 in methylator colon cancers. *Br. J. Cancer* **119**, 1267–1277 (2018). 30318507 10.1038/s41416-018-0249-9 PMC6251029

[19] Zhang, B. et al. Proteogenomic characterization of human colon and rectal cancer. *Nature* **513**, 382–387 (2014). 25043054 10.1038/nature13438 PMC4249766

[20] Li, X. et al. A modified protein marker panel to identify four consensus molecular subtypes in colorectal cancer using immunohistochemistry. *Pathol. -Res. Pract.* **220**, 153379 (2021). 33721619 10.1016/j.prp.2021.153379

[21] Clarke, C. N. et al. Proteomic features of colorectal cancer identify tumor subtypes independent of oncogenic mutations and independently predict relapse-free survival. *Ann. Surg. Oncol.* **24**, 4051–4058 (2017). 28936799 10.1245/s10434-017-6054-5 PMC6063735

[22] Kanehisa, M. & Goto, S. Kegg: Kyoto encyclopedia of genes and genomes. *Nucleic Acids Res.* **28**, 27–30 (2000). 10592173 10.1093/nar/28.1.27 PMC102409

[23] Kanehisa, M., Furumichi, M., Sato, Y., Kawashima, M. & Ishiguro-Watanabe, M. Kegg for taxonomy-based analysis of pathways and genomes. *Nucleic Acids Res.* **51**, D587–D592 (2023). 36300620 10.1093/nar/gkac963 PMC9825424

[24] Subramanian, A. et al. Gene set enrichment analysis: a knowledge-based approach for interpreting genome-wide expression profiles. *Proc. Natl Acad. Sci.* **102**, 15545–15550 (2005). 16199517 10.1073/pnas.0506580102 PMC1239896

[25] Liberzon, A. et al. The molecular signatures database hallmark gene set collection. *Cell Syst.* **1**, 417–425 (2015). 26771021 10.1016/j.cels.2015.12.004 PMC4707969

[26] Huang, J. et al. Unfolded protein response in colorectal cancer. *Cell Biosci.* **11**, 1–16 (2021). 33514437 10.1186/s13578-021-00538-z PMC7844992

[27] Kawaguchi, J. et al. Cisplatin and ultraviolet-C synergistically down-regulate receptor tyrosine kinases in human colorectal cancer cells. *Mol. Cancer* **11**, 1–11 (2012). 22788833 10.1186/1476-4598-11-45 PMC3477093

[28] Bates, R. C. & Mercurio, A. The epithelial-mesenchymal transition (EMT) and colorectal cancer progression. *Cancer Biol. Ther.* **4**, 371–376 (2005). 15846061 10.4161/cbt.4.4.1655

[29] Zhang, N. et al. Novel therapeutic strategies: Targeting epithelial–mesenchymal transition in colorectal cancer. *Lancet Oncol.* **22**, e358–e368 (2021). 34339656 10.1016/S1470-2045(21)00343-0

[30] Stark, G. R. & Taylor, W. R. Analyzing the g2/m checkpoint. *Checkpoint Controls and Cancer: Volume 1: Reviews and Model Systems* 51–82 (2004). 10.1385/1-59259-788-2:051 15187249

[31] Csurka, G., Dance, C., Fan, L., Willamowski, J. & Bray, C.*Visual categorization with bags of keypoints*, 1–22 (Springer, Prague, Czech Republic, 2004).

[32] Sivic, J. & Zisserman, A. *Video Google: A text retrieval approach to object matching in videos*, 1470–1477 (IEEE, 2003).

[33] Bouslimi, R., Messaoudi, A. & Akaichi, J. Using a bag of words for automatic medical image annotation with a latent semantic. *Int. J. Artif. Intell. Appl.* **4**, 51 (2013).

[34] Edwards, N. J. et al. The CPTAC data portal: a resource for cancer proteomics research. *J. Proteome Res.* **14**, 2707–2713 (2015). 25873244 10.1021/pr501254j

[35] Marisa, L. et al. Intratumor cms heterogeneity impacts patient prognosis in localized colon cancer. *Clin. Cancer Res.* **27**, 4768–4780 (2021). 34168047 10.1158/1078-0432.CCR-21-0529 PMC8974433

[36] Thanki, K. et al. Consensus molecular subtypes of colorectal cancer and their clinical implications. *Int. Biol. Biomed. J.* **3**, 105 (2017). 28825047 PMC5557054

[37] Oliveira, C. et al. Kras and Braf oncogenic mutations in MSS colorectal carcinoma progression. *Oncogene* **26**, 158–163 (2007). 16953233 10.1038/sj.onc.1209758

[38] Smeby, J. et al. CMS-dependent prognostic impact of Kras and brafv600e mutations in primary colorectal cancer. *Ann. Oncol.* **29**, 1227–1234 (2018). 29518181 10.1093/annonc/mdy085 PMC5961317

[39] Network, C. G. A. et al. Comprehensive molecular characterization of human colon and rectal cancer. *Nature* **487**, 330 (2012). 22810696 10.1038/nature11252 PMC3401966

[40] Jiang, X. et al. End-to-end prognostication in colorectal cancer by deep learning: a retrospective, multicentre study. *Lancet Digit. Health* **6**, e33–e43 (2024). 38123254 10.1016/S2589-7500(23)00208-X

[41] Macenko, M. et al. *A method for normalizing histology slides for quantitative analysis*, 1107–1110 (IEEE, 2009).

[42] Dosovitskiy, A. et al. An image is worth 16 × 16 words: Transformers for image recognition at scale. In *International Conference on Learning Representations* (Austria, 2021).

[43] Pedregosa, F. et al. Scikit-learn: Machine learning in Python. *J. Mach. Learn. Res.* **12**, 2825–2830 (2011).

[44] Satopaa, V., Albrecht, J., Irwin, D. & Raghavan, B.*Finding a “kneedle” in a haystack: Detecting knee points in system behavior*, 166–171 (IEEE, 2011).

[45] Arthur, D. & Vassilvitskii, S. k-means++: The advantages of careful seeding. Tech. Rep., Stanford (2006).

[46] Lloyd, S. Least squares quantization in PCM. *IEEE Trans. Inf. theory* **28**, 129–137 (1982).

[47] Kaplan, E. L. & Meier, P. Nonparametric estimation from incomplete observations. *J. Am. Stat. Assoc.* **53**, 457–481 (1958).

[48] David, G. K. & Mitchel, K. *Survival Analysis: A Self-Learning Text*, 3rd ed. (Springer, 2012).

[49] Stelzer, G. et al. The genecards suite: from gene data mining to disease genome sequence analyses. *Curr. Protoc. Bioinforma.* **54**, 1–30 (2016). 10.1002/cpbi.5 27322403

[50] Safran, M. et al. The Genecards suite.In *Practical guide to life science databases*, 27–56 (Springer Nature Singapore, Singapore, 2022).

[51] Goldman, M. J. et al. Visualizing and interpreting cancer genomics data via the Xena platform. *Nat. Biotechnol.* **38**, 675–678 (2020). 32444850 10.1038/s41587-020-0546-8 PMC7386072

[52] Vejnar, C. E. & Zdobnov, E. M. Mirmap: comprehensive prediction of microRNA target repression strength. *Nucleic Acids Res.* **40**, 11673–11683 (2012). 23034802 10.1093/nar/gks901 PMC3526310

[53] Russo, F. et al. Mirandola 2017: a curated knowledge base of non-invasive biomarkers. *Nucleic Acids Res.* **46**, D354–D359 (2018). 29036351 10.1093/nar/gkx854 PMC5753291

