# Supplementary material for: Multi-modal clustering reveals event-free patient subgroup in colorectal cancer survival
Source: NPJ Syst Biol Appl. 2025 Aug 2;11:86. doi: 10.1038/s41540-025-00557-3 (PMC12318085; doi:10.1038/s41540-025-00557-3)
Supplement: Supplementary file 1 — Supplementary information [file 41540_2025_557_MOESM1_ESM.pdf]

Supplementary Information for Multi-modal clustering reveals event-free patient subgroup in colorectal cancer survival

Note S1 Multi-omics signature

This section lists the features that are used in our analyses to compose the multi-omics signature.

Note S1.1 Gene expression

The 40 genes selected from [1] are as follows: AXIN2, SEMA5A, CDHR1, ASPHD2, CTTNBP2, WARS1, HPSE, ATP9A, GNLY, DACH1, POFUT1, QPRT, PLAGL2, LYZ, TP53RK, CEL, TRIB2, DDX27, DUSP4, FSCN1, TIMP3, CAPN9, VAV2, RBMS1, B3GNT6, FCGBP, RASD1, ASRGL1,SPINK4, FBN1, ARMCX1, COL8A2, RAB34, MGP, TGFB3, AOC3, TNS1, CCDC80, SDC2, and NRP2.

Note S1.2 Protein expression

The 11 proteins that make up the signature are: BETACATENIN [2, 3], P53 [2], COLLAGENVI, FOXO3A, INPP4B, PEA15, PRAS40PT246, RAD51, S6, S6PS235S236, and S6PS240S244 [4].

Note S1.3 miRNA expression

The 30 miRNAs that make up the signature are: hsa-mir-99a, hsa-mir-100 [5], hsa-mir-1247 [6, 7], hsa-mir-10a, hsa-mir-20a, hsa-mir-27a, hsa-mir-92a-1, hsa-mir-92a-2, hsa-mir-486-1, hsa-mir-486-2, hsa-mir-584 [8], hsa-mir-144, hsa-mir-425, hsa-mir-483 [7, 9, 10], hsa-mir-221, hsa-mir-222, hsa-mir-223, hsa-mir-135b, hsa-mir-141, hsa-mir-143, hsa-mir-145, hsa-mir-200a, hsa-mir-200b, hsa-mir-200c, hsa-mir-15b, hsa-mir-17, hsa-mir-18a, hsa-mir-19a, hsa-mir-21, and hsa-mir-29a [11].

Note S1.4 DNA methylation

80 probes were found in common between the TCGA COADREAD dataset and literature shortlisted DNA methylation signatures. The probes are listed as follows:

1. Aberrantly methylated genes mapped to probes [12, 13] - cg22459146, cg23418465, cg02310296, cg17795240, cg07799947, cg25216696, cg06274159, cg12842316,

cg05970790, cg16744741, cg14189571, cg24646414, cg19523029, cg19172575, cg26680127, cg01546563, cg09626984, cg14383135, cg21073927, cg26020513, cg14256699, cg08460026, cg13247990, cg11812218, cg00707317, cg18689253, cg05801573, cg10202457, cg06973760, cg20925811, cg18904346, cg13434842, cg20279283, cg14093936, cg18123948, cg14443380, cg13915726, cg10866709, cg22264436, cg00090147, cg25057743, and cg11981599.
2. Epigenome-wide paired DNA methylation profiling [14] - cg06786372, cg17301223, cg15638338, cg18065361.
3. DNA methylation signature from peripheral blood [15] - cg24702253, cg06551493, cg12691488, cg17292758, cg16170495, cg21585512, cg17187762, cg05983326, cg11885357.
4. DNA methylation markers that distinguish CMS2 and CMS3 [16] - cg23928468, cg00901574, cg00512872, cg17842966, cg00097384, cg00145955, cg27603796, cg16708174, cg20698769, cg23045908, cg04739880, cg19107055, cg17477990, cg00901138, cg05951860, cg16477879, cg19335412, cg12492273, cg23219253, cg14754494, cg11125249, cg16772998, cg02827572, cg05211192, cg05357660.

Note S2 Extended multi-modal results

Table S1: Number of missing values from each modality after outer-join.

Genes	CpG Sites	Proteins	miRNAs
15	236-238	167	32

Note S2.1 Cluster stability

The K-means algorithm is initialised with the K-means++ method to stabilise clustering to a certain degree. Nevertheless, since the first centroid of the K-means++ algorithm is chosen at random, we further assess the stability of clusters generated by multi-omics and multi-modal data by repeating the clustering 5 times, each time with a different seed. We find that the clusters are stable (optimal number of clusters=4) and an all-surviving cluster is identified in all cases. We report this analysis in Figure S1 for multi-omics data and Figure S2 for multi-modal data.

Note S2.2 Feature importance

For brevity, we show the top 10 discriminating features for each cluster pair in Figure S5. We find less overlap of features between the cluster pairs generated by multi-omics than single omics (Note S3.1). This is a good indicator of cluster separability and improves cluster characterisation. We see similar results for multi-modal clusters in Figure S6.

Table S2: Average silhouette scores for various clusters K. We compute this score to verify the quality of the optimal number of clusters as suggested by the elbow method (highlighted in bold).

Modality	K=2	K=3	K=4	K=5	K=6	K=7	K=8
Protein Expression	0.153726	0.125567	**0.111632**	0.112769	0.105113	0.110578	0.095420
DNA Methylation	0.233902	0.112777	**0.122207**	0.082718	0.065518	0.055676	0.053181
miRNA Expression	0.156211	0.092768	0.103739	**0.103316**	0.094286	0.093441	0.088001
Whole Slide Images	0.241252	0.196735	**0.154045**	0.144498	0.148724	0.150276	0.139844
Gene Expression	0.202771	0.152757	**0.135089**	0.128523	0.111609	0.116389	0.105391
Multi-omics	0.174319	0.140909	**0.067815**	0.072879	0.052132	0.071308	0.045142
Multi-modal	0.173084	0.138951	**0.076185**	0.075296	0.044934	0.066791	0.030503

Table S3: Number of missing values from the top 10 discriminatory features between cluster pairs identified by multi-omics data.

**Feature Type**	**Count**	**Features**
Genes	15	AOC3, ATP9A, AXIN2, CCDC80, CDHR1, COL8A2, DUSP4, FBN1, MGP, NRP2, PLAGL2, POFUT1, QPRT, RAB34, SEMA5A, TGFB3, TNS1, TRIB2
miRNAs	32	hsa-mir-100, hsa-mir-141, hsa-mir-200a
CpG Sites	236	cg00512872, cg00901574, cg02827572, cg05211192, cg11125249, cg12492273, cg14754494, cg16477879, cg16708174, cg16772998, cg17477990, cg19107055, cg23219253, cg23928468

Note S2.3 Multi-omics results with inner-join

A binary heatmap of the union of the top 10 features that discriminate between the clusters is shown in Figure S8.

The union of the top 10 features that discriminate each cluster pair results in 37 unique features for the inner-joined multi-omics data, compared to 35 unique features for the outer-joined multi-omics data. There are 24 features in common between the two cases, which are: 'AOC3', 'AXIN2', 'CCDC80', 'COL8A2', 'DUSP4', 'FBN1', 'MGP', 'QPRT', 'TGFB3', 'TNS1', 'cg00512872', 'cg00901574', 'cg02827572', 'cg05211192', 'cg11125249', 'cg12492273', 'cg16477879', 'cg16708174', 'cg16772998', 'cg19107055', 'cg23219253', 'cg23928468', 'hsa-miR-100', and 'hsa-miR-141'.

Note S2.4 Correlation between omics and whole slide image features

To determine the existence of redundancies, we first compute the pairwise sample distances using the cosine metric in both the omics and image space, following which we compute the Pearson correlation between the flattened lower triangular distance matrices of the two spaces, which resulted in no correlations (`pearsonr` statistic=0.093, pvalue=7.51e-127). We also perform a `pearsonr` pairwise correlation analysis directly

on the features. We only select patients who have all modalities recorded. For better figure readability given the large number of features, we set an absolute correlation threshold of 0.3 such that only pairs of features with absolute correlation greater than this threshold are highlighted in the heatmap shown in Figure S9. We see little to no correlation between the omics features and image features. Given the large number of features, not all feature names are annotated in the figure. The order of features is omics first, followed by image features.

Note S2.5 Clinico-pathological characterisation of clusters

We extend the analysis of clusters beyond disease-specific survival to other clinical variables to - (i) identify any confounding effects from known factors such as age and gender, and (ii) obtain a more insightful characterisation of the clusters. We study the per-cluster distributions of the following clinical variables: age, weight, gender, stage, tumour site, presence of colon polyps, BRAF abnormality, KRAS mutation, venous invasion, microsatellite instability (MSI), CpG-island methylator phenotype (CIMP), and CMS. The chi-square test is used to determine whether the differences between clusters with respect to these clinical variables are significant.

Both multi-omics and multi-modal clustering reveal similar distributions of clinical variables between the clusters. The significance of differences between the clusters with respect to the clinical variables under study is shown in Table S4 for multi-omics clusters and Table S5 for multi-modal clusters. Confounding variables like age and gender have no effect on the clusters (Figure S10, Figure S16). Tumour stage (Figure S13, Figure S19) is distinctly distributed between the clusters, with the all-surviving cluster having majority stage i and stage ii cases. Tumour location is also markedly different, primarily located in the ascending colon and cecum in the all-surviving cluster, which also shows no venous invasion, another attribute that significantly differs between the clusters (Figure S14, Figure S20). The clusters also significantly differ in MSI and CIMP status, with the all-surviving cluster being primarily MSI-high and CIMP-high (Figure S12,Figure S18). The presence of an abnormal BRAF gene is another differentiating factor of the all-surviving cluster (Figure S11,Figure S17). Interestingly, the presence of KRAS mutations (Figure S11, Figure S17) and colon polyps (Figure S13, Figure S19) have no effect on the clusters.

Survival of CRC patients is impacted by the complex interplay between BRAF mutation, KRAS mutation, MSI, and CIMP [17, 18]. This interplay is reflected in the clusters identified by multi-omics signature. For instance, high levels of genomic instability, which can have variable effects on mutations that either improve or worsen survival outcomes [19–21], have been observed in both the all-surviving cluster and the poor 10-year survival cluster. This multi-omics approach offers advantages over consensus molecular subtypes (CMS), which assign each subtype to a specific phenotype. For example, CMS1 is characterized by high MSI and worse survival after relapse, and CMS2 is associated with the best prognosis [22]. When examining disease-specific survival, however, these CMS classifications don't fully capture the heterogeneity in patient outcomes due to various factors, including genomic instability. An observation of the distribution of CMS in the multi-modal clusters (Figure S15) reveals that CMS1 patients are not only found in the poor 10-year survival cluster but also in the

all-surviving cluster owing to its high microsatellite instability. On the contrary, there are no CMS2 patients in the all-surviving cluster, despite its positive association with survival. Thus, our multi-omics clustering reveals more nuanced survival patterns that are not evident in the CMS framework.

We further analyse the differences specifically between the all-surviving and poor 10-year survival clusters. Both clusters are largely similar in the distribution of most clinical variables. We find that only stage and venous invasion are significantly different between these 2 clusters, which are captured by multi-omics data.

Table S4: Comparison of chi-squared test statistic p-values between all multi-omics clusters and poor 10-year cluster2 vs all-surviving cluster3.

Feature	p-values (all)	p-values (cluster2 vs cluster3)
Age	0.3209	0.84505
Gender	0.2600	0.7673
BRAF Gene Status	0.0012	0.3241
KRAS Mutation	0.5564	0.5186
MSI	5.07e-25	0.2256
CIMP	1.92e-13	0.1267
Stage	3.32e-05	8.63e-03
Colon Polyps	0.6201	0.8933
Tumour Location	3.29e-09	0.6352
Venous Invasion	0.0153	0.0056
CMS	7.26e-75	1.75e-14

Table S5: Comparison of chi-squared test statistic p-values between all multi-modal clusters and poor 10-year cluster1 vs all-surviving cluster0.

Feature	p-values (all)	p-values (cluster0 vs cluster1)
Age	0.3209	0.84505
Gender	0.2632	1.0000
BRAF Gene Status	0.0012	0.3241
KRAS Mutation	0.5452	0.5186
MSI	8.04e-23	0.2329
CIMP	1.55e-12	0.0965
Stage	2.38e-05	0.0075
Colon Polyps	0.5012	0.9307
Tumor Location	4.44e-08	0.5775
Venous Invasion	0.0139	0.0073
CMS	1.67e-71	3.16e-15

Note S2.6 Bag-of-visual-words representation of whole slide images

Reducing whole slide images to the mean of all patch representations could result in loss of local information. Given the heterogeneity of tumours, we consider another

summarisation approach based on bag-of-words to assess the impact on clustering, if any. We implement a bag-of-visual-words approach [23–25] and summarise the patches by two metrics - normalised counts and Term Frequency-Inverse Document Frequency (TF-IDF). The images are processed into patches by the pipeline described in Section ?? of the main manuscript. Each patch is a 768-dimensional embedding and is independently normalised before fitting a `K-means` model as implemented by `scikit-learn` [26] on it. The patches are normalised to ensure the cosine metric is used to compute distances. The number of clusters determines the representation size of the image. To this end, we consider k = [8,16,32,64,128] clusters and pick the optimal number of clusters k=32 using the elbow method following the Kneedle algorithm [27]. Each patch is assigned to a cluster. The patches are then grouped by patient ID and aggregated by counts. This leaves us with a count matrix, where each row is a 32-dimensional vector for a given patient, each column represents a cluster ID, and the value is the number of patches of a given patient assigned to a given cluster.

Note S2.6.1 Normalised counts

The count matrix is normalised per patient and merged with multi-omics data using an outer-join. This normalised count multi-modal dataset is then median-imputed to fill any missing values per feature. We repeat the analysis outlined in Section ?? of the main manuscript on this dataset. The results are consistent with our findings on the multi-modal dataset - optimal number of clusters is k=4 (Figure S21a)), and there exists an all-surviving cluster (cluster 3) and a cluster with poor 10-year survival (cluster 0) (Figure S23a)). The Peto-weighted log-rank test p-values for the clusters are reported in Table S6. The top 10 features as identified by ANOVA are shown in Figure S24a), which also shows similarities to Figure S6. A gene set enrichment analysis reveals the same 4 pathways as being enriched - (i) Unfolded Protein Response (adjusted p-value 0.0405), (ii) UV Response Downregulation (adjusted p-value 0.0405), (iii) Epithelial Mesenchymal Transition (EMT) (adjusted p-value 0.0418) and, (iv) G2-M Checkpoint (adjusted p-value 0.04118). A correlation study of inter-patient distances between omics and normalised count bag-of-patches reveals little correlation (`pearsonr`statistic=0.1764, p-value=0.0).

Note S2.6.2 TF-IDF

The count matrix is transformed to a normalised TF-IDF vector using the transformation function made available by `scikit-learn` [26]. This normalised TF-IDF multi-modal dataset is then median-imputed to fill any missing values per feature. The same process is repeated as above. The optimal number of clusters is k=4 (Figure S21b)), and there exists an all-surviving cluster (cluster 0) (Figure S23b)). However, we no longer see a cluster where the 10-year survival probability drops to 0. The Peto-weighted log-rank test p-values for the clusters are reported in Table S6. The top 10 features as identified by ANOVA are shown in Figure S24b), which also shows similarities to Figure S6. A gene set enrichment analysis reveals the same 4 pathways as being enriched - (i) Unfolded Protein Response (adjusted p-value 0.0405), (ii) UV Response Downregulation (adjusted p-value 0.0405), (iii) Epithelial Mesenchymal Transition (EMT) (adjusted p-value 0.0418) and, (iv) G2-M Checkpoint (adjusted

p-value 0.04118). A correlation study of inter-patient distances between omics and normalised count bag-of-patches reveals little correlation (`pearsonr` statistic=0.1750, p-value=0.0).

Table S6: Comparison of p-values computed using Peto-weighted log-rank statistics across different modalities to identify clusters that are significantly different in disease-specific survival. Significant p-values < 0.05 are highlighted in bold.

**Normalised count multi-modal data**			**Normalised TF-IDF multi-modal data**		
$cluster_i$	$cluster_j$	$p - value$	$cluster_i$	$cluster_j$	$p - value$
0	1	0.11	0	1	**0.01**
0	2	0.20	0	2	< **0.005**
0	3	< **0.005**	0	3	**0.01**
1	2	0.56	1	2	0.33
1	3	**0.01**	1	3	0.51
2	3	**0.01**	2	3	0.19

Note S2.7 Proxy study on CPTAC using multi-omics

In this study, we validate the multi-omics signatures and clusters derived from TCGA on the CPTAC dataset [28]. The CPTAC dataset comprises RNASeq, miRNA expression, and overall survival information for colon cancer. We disregard the proteomic data as the technology used to measure it (Mass Spectrometry) differs from that used in TCGA (RPPA). The CPTAC dataset is created by an outer-join of the two available modalities and is standardised and imputed based on TCGA's parameters. As CPTAC does not have all modalities recorded by TCGA, we use a proxy to retrieve cluster membership of CPTAC patients. We set up the proxy as follows:

1. Subset TCGA data to include only RNASeq and miRNA features available for CPTAC.
2. Retrieve cluster labels for TCGA patients.
3. Fit a K-Nearest Neighbours Classifier model (k=5) on this TCGA data using the cosine metric to compute distances.
4. Predict on CPTAC data to get cluster assignments.

Since DSS information is not available for CPTAC, we plot the Kaplan-Meier curves for the CPTAC clusters using overall-survival (OS) information (Figure S25). We see similar trends as with TCGA data - an all-surviving cluster (albeit with only 1 patient) and a cluster having survival probability 0 after approximately 45 months. The Peto-weighted log-rank test did not yield statistical significance between the different survival functions (Table S7).

Table S7: Comparison of p-values computed using Peto-weighted log-rank statistics between CPTAC multi-omics clusters using overall survival to compute the survival function. No significant differences (p-value < 0.05) are found.

$cluster_i$	$cluster_j$	$p - value$
0	1	0.65
0	2	0.07
0	3	0.83
1	2	0.23
1	3	0.80
2	3	0.64

Note S3 Unimodal results

Although DNA methylation is able to identify a cluster of patients who experience no event at all (Figure S28a)), the difference is significant compared to the other clusters only when other omics is included, i.e, in the multi-omics setting.

Note S3.1 Feature importance

The union of the top 10 features for each cluster pair is visualised with a binary heatmap to qualitatively assess the degree of overlap. We find significant overlap for miRNA (Figure S31) and gene expression (Figure S32). DNA methylation shows relatively less overlap (Figure S29, and protein expression shows the most simply because it only has 11 features in total (Figure S30).

Note S4 Distribution of OS across clusters

The results from the Kaplan-Meier plots are similar to what we see with DSS. This is expected as OS encompasses DSS and other causes for death. From the unimodal results (Figure S34), only gene expression and DNA methylation values seem to produce clusters with a significantly different survival function. In DNA methylation (Figure S34a)), we see that cluster 1 is almost exclusively made up of 0-event patients, save 2. This is the only cluster with a significantly different survival rate ($p = 0.05$) compared to cluster 0 as measured by the Peto-weighted pairwise log-rank test. Gene expression-based clustering produces the best results among the unimodal datasets (Figure S33c)), despite the absence of clusters unique to an event. From a Peto weighted pairwise log-rank test, we find that cluster 3 is significantly different compared to cluster 1 ($p = 0.04$) and cluster 2 ($p < 0.005$), and cluster 2 is significantly different compared to cluster 0 ($p = 0.03$).

With multi-omics based clustering (Figure S33b)), cluster 3 has the lowest number of deaths (=5), and it is significantly better in terms of survival than cluster 0 ($p = 0.05$), cluster 1 ($p = 0.05$) and cluster 2 ($p < 0.005$). Cluster 2 is another cluster identified by multi-omics as being significantly worse than cluster 0 ($p = 0.04$), cluster 1 ($p = 0.03$) and cluster 3 ($p < 0.005$).

Multi-modal clustering (Figure S33a)) does not seem to improve much over multi-omics clustering. Cluster 0, with the lowest number of deaths, is significantly better than cluster 1 ($p < 0.005$) and cluster 3 ($p = 0.03$) in terms of survival, and cluster 1 is significantly different in survival compared to cluster 2 ($p = 0.03$). For the CMS-based clusters, the Peto-weighted pairwise log-rank test does not identify any clusters that are significantly different from one another.

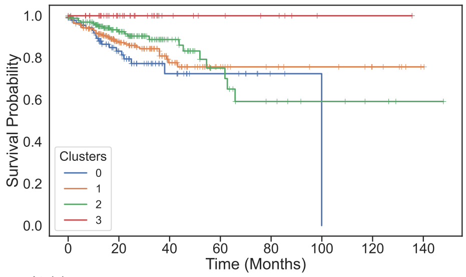

At risk								
Cluster 0	90	46	15	8	2	0	0	0
Cluster 1	272	144	45	19	13	10	6	1
Cluster 2	169	98	36	15	8	5	3	1
Cluster 3	47	28	10	7	5	1	1	0

a) Random state 1450.

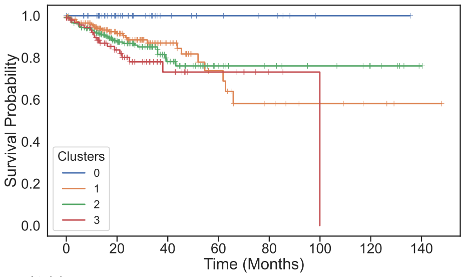

At risk								
Cluster 0	47	28	10	7	5	1	1	0
Cluster 1	178	101	38	15	8	5	3	1
Cluster 2	260	139	43	19	13	10	6	1
Cluster 3	93	48	15	8	2	0	0	0

b) Random state 649.

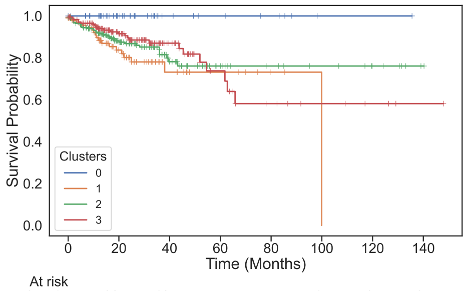

At risk								
Cluster 0	47	28	10	7	5	1	1	0
Cluster 1	93	48	15	8	2	0	0	0
Cluster 2	260	139	43	19	13	10	6	1
Cluster 3	178	101	38	15	8	5	3	1

c) Random state 2885.

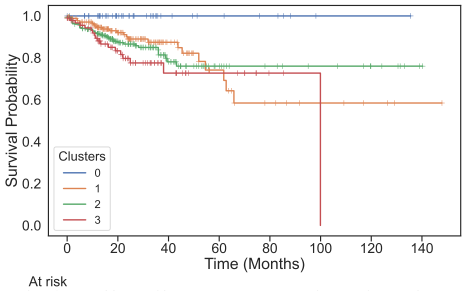

At risk								
Cluster 0	47	28	10	7	5	1	1	0
Cluster 1	176	100	37	15	8	5	3	1
Cluster 2	264	141	44	19	13	10	6	1
Cluster 3	91	47	15	8	2	0	0	0

d) Random state 3467.

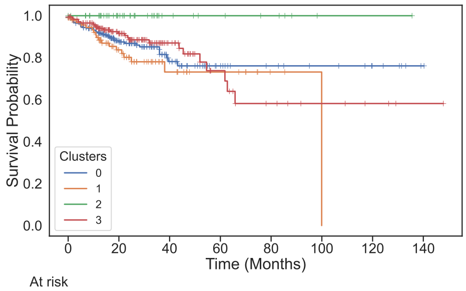

At risk								
Cluster 0	260	139	43	19	13	10	6	1
Cluster 1	93	48	15	8	2	0	0	0
Cluster 2	47	28	10	7	5	1	1	0
Cluster 3	178	101	38	15	8	5	3	1

e) Random state 3633.

Fig. S1: **Cluster-stability of multi-omics data.** Kaplan-Meier plots of disease-specific survival (DSS) across multi-omics clusters found by different seeds. The clusters are stable with different initial centroids.

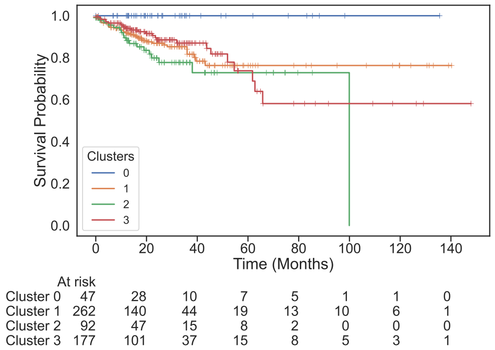

a) Random state 1450.

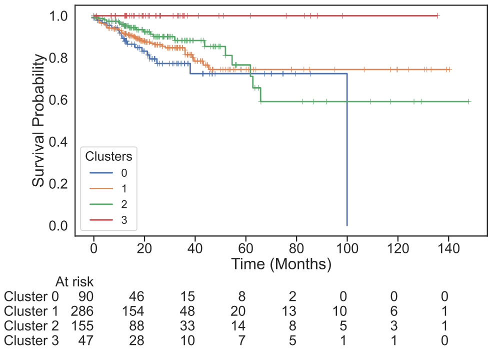

b) Random state 649.

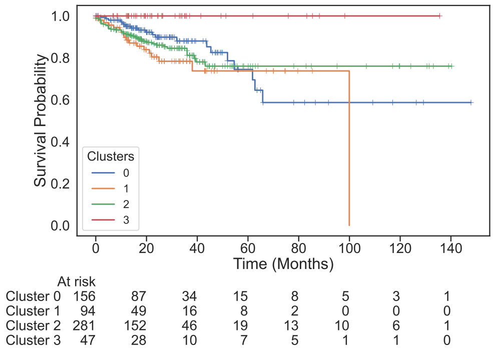

c) Random state 2885.

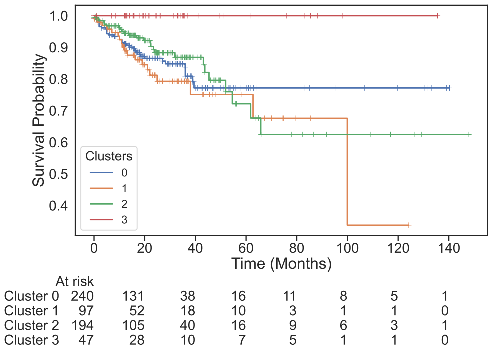

d) Random state 3467.

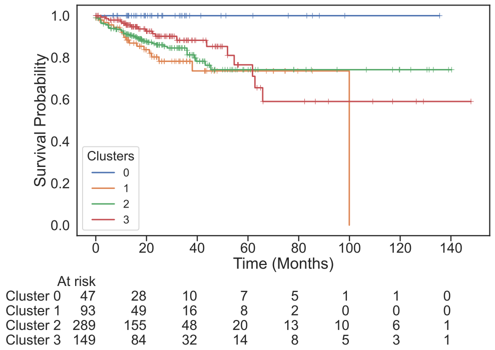

e) Random state 3633.

Fig. S2: **Cluster-stability of multi-modal data.** Kaplan-Meier plots of disease-specific survival (DSS) across multi-modal clusters found by different seeds. The clusters are stable with different initial centroids.

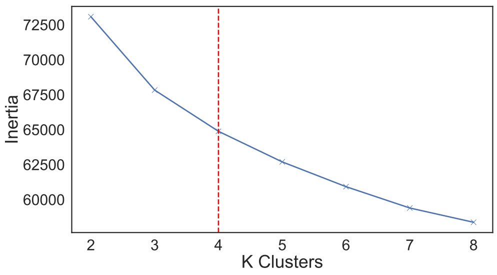

a) Multi-modal dataset.

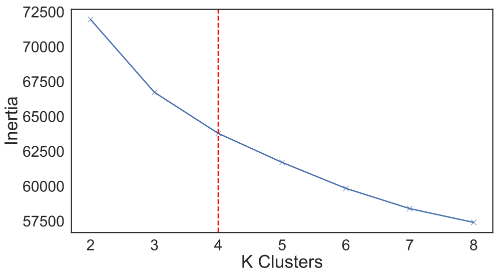

b) Multi-omics dataset.

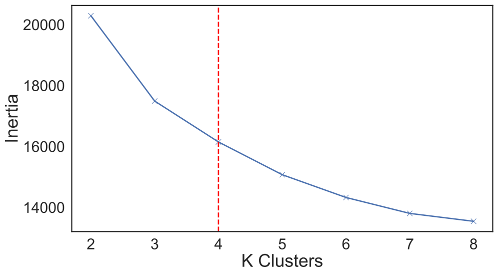

c) Gene expression.

Fig. S3: **Optimal number of clusters $K = 4$.** For each dataset, the optimal number of clusters is found using the elbow method by plotting the sum of squared errors (inertia) against the number of clusters.

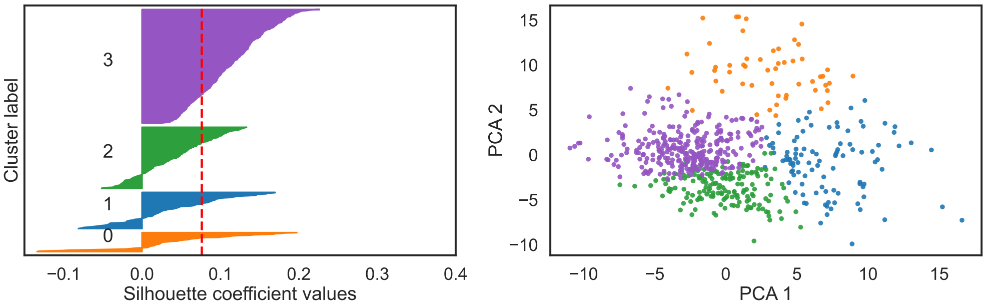

a) Multi-modal dataset.

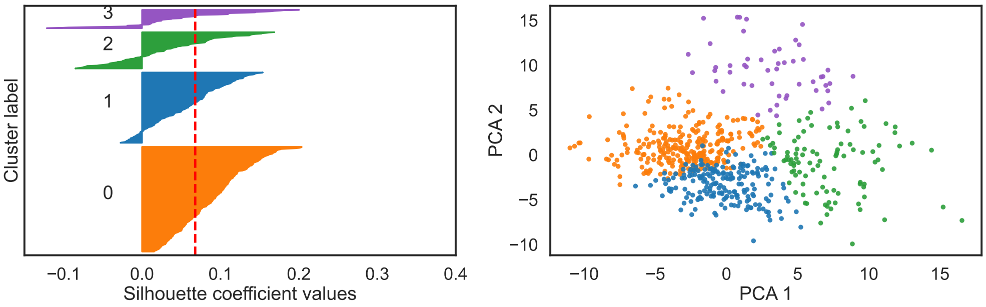

b) Multi-omics dataset.

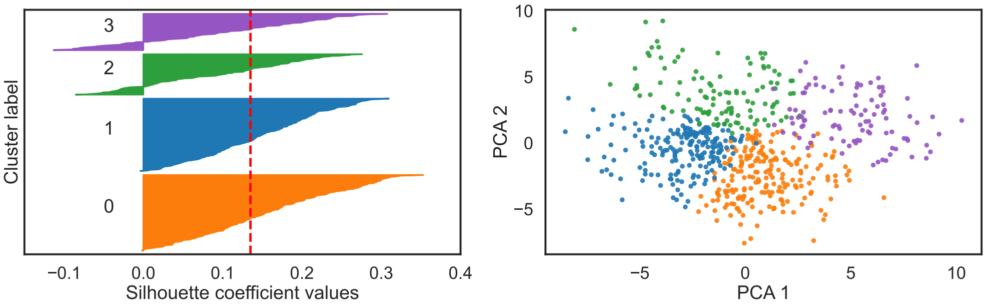

c) Gene expression.

Fig. S4: **PCA visualisation of the optimal number of clusters $K = 4$.** For each dataset, clustering is performed with the optimal number of clusters and visualised with principal component analysis. Additionally, a silhouette analysis is carried out to measure the tightness of the clusters.

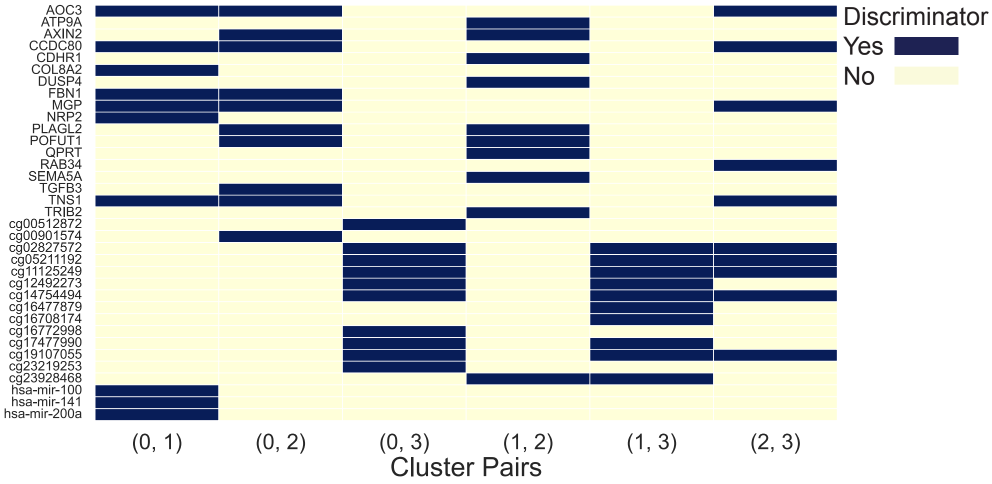

Fig. S5: Feature importance binary heatmap for outer-joined multi-omics dataset. The union of the top 10 features for each cluster pair is visualised against the cluster pair, enabling qualitative assessment of the degree of overlap.

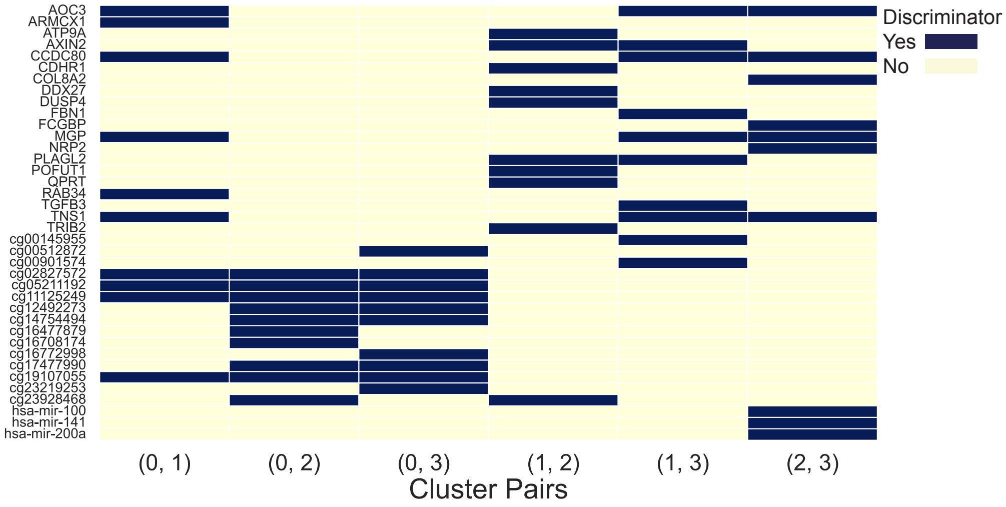

Fig. S6: Feature importance binary heatmap for outer-joined multi-modal dataset. The union of the top 10 features for each cluster pair is visualised against the cluster pair, enabling qualitative assessment of the degree of overlap.

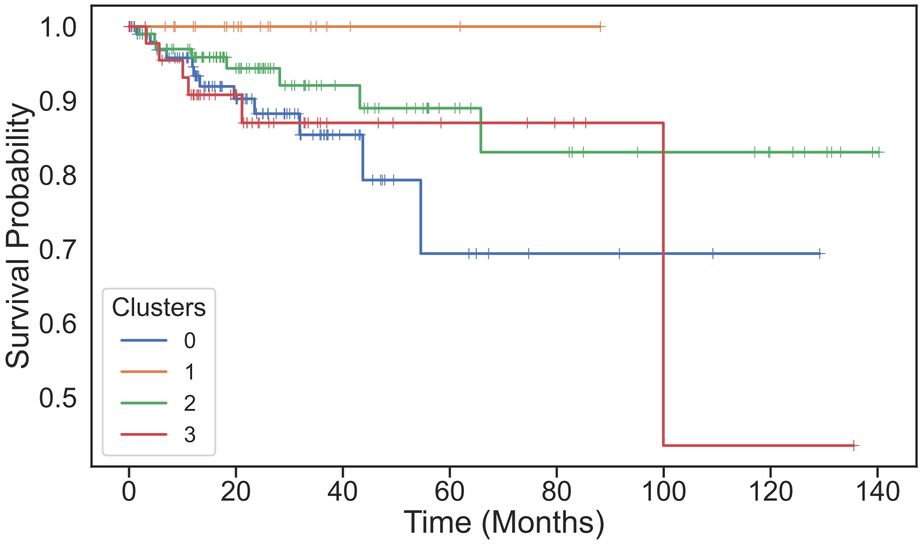

At risk								
Cluster 0	99	54	17	7	3	2	1	0
Cluster 1	21	12	3	2	1	0	0	0
Cluster 2	103	63	30	18	14	10	7	1
Cluster 3	47	25	9	6	4	1	1	0

Fig. S7: Multi-omics signatures of patients with all modalities recorded also identify an all-surviving cluster, ruling out any false positives due to imputation artefacts.

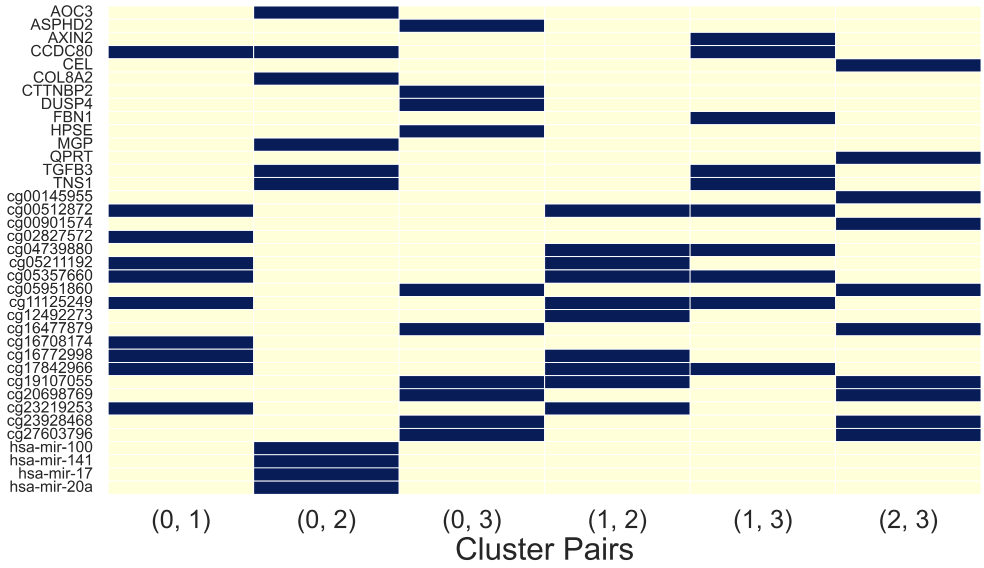

Fig. S8: Feature importance binary heatmap for inner-joined multi-omics dataset. The union of the top 10 features for each cluster pair is visualised against the cluster pair to which it contributes to qualitatively asses degree of overlap.

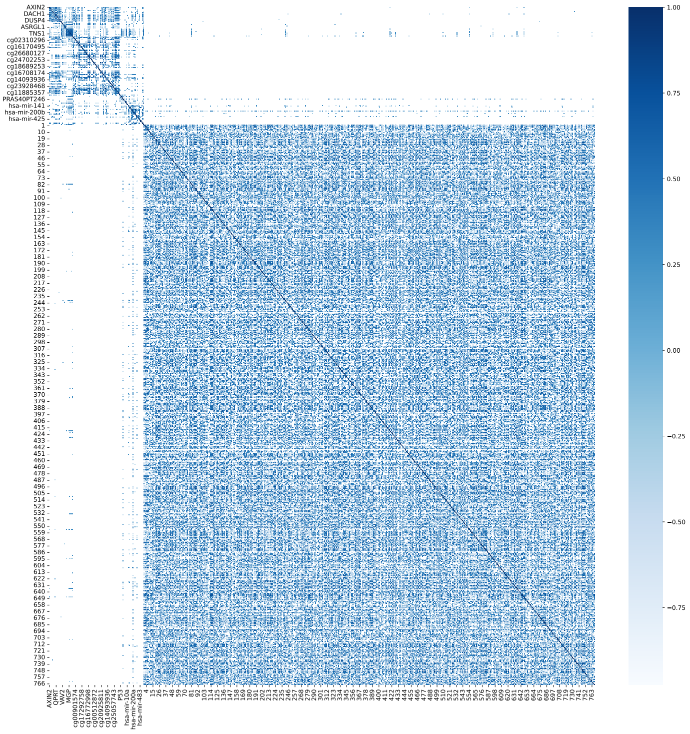

Fig. S9: Pairwise `pearsonr` correlation between multi-modal features. The heatmap highlights all features with absolute correlation greater than 0.3. We see little to no correlation between the omics features and image features.

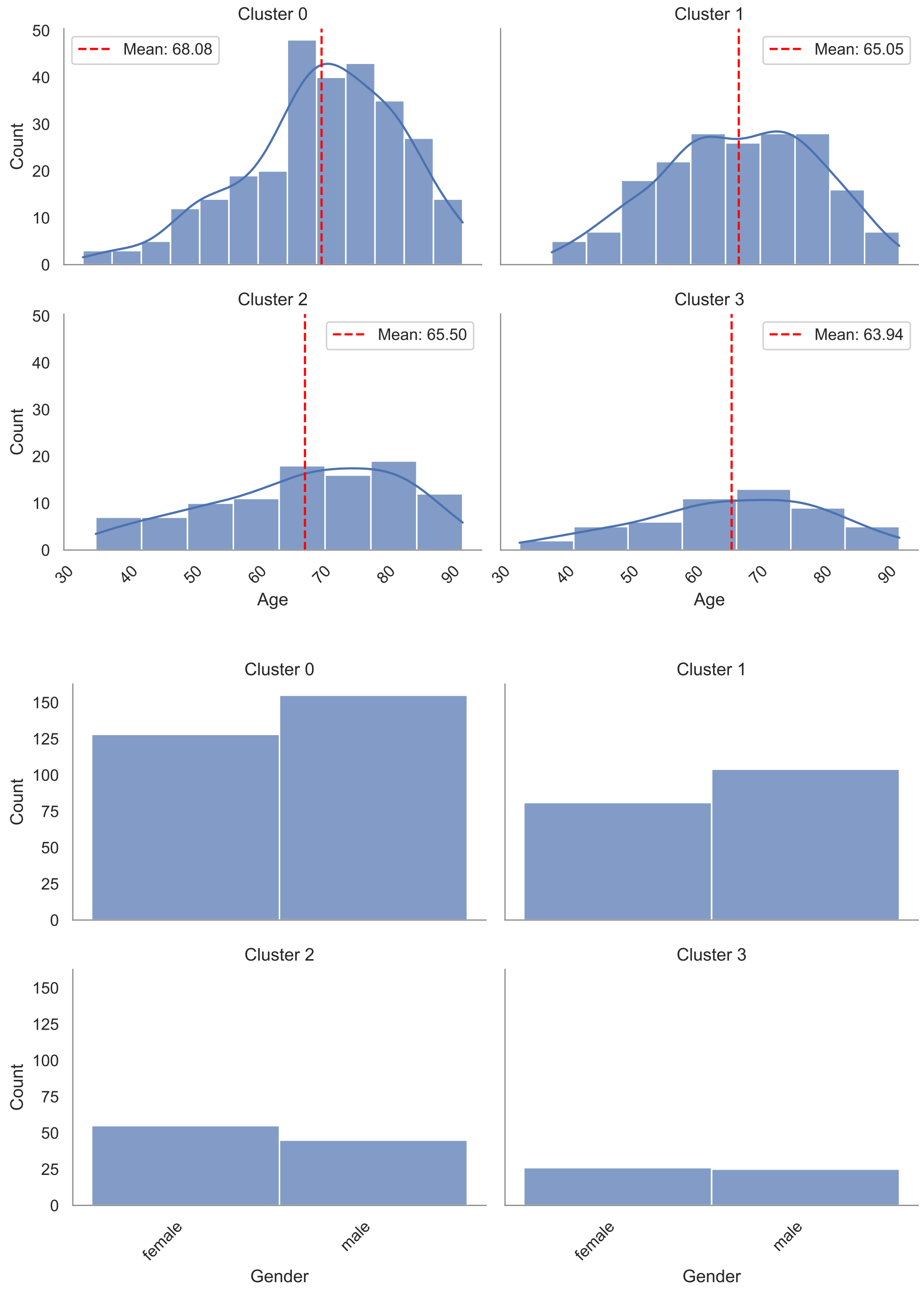

Fig. S10: **Distribution of age and gender in clusters generated by multi-omics data.** There is no bias towards key confounding factors such as age and gender on disease-specific survival in the all-surviving cluster 3 identified by multi-omics data.

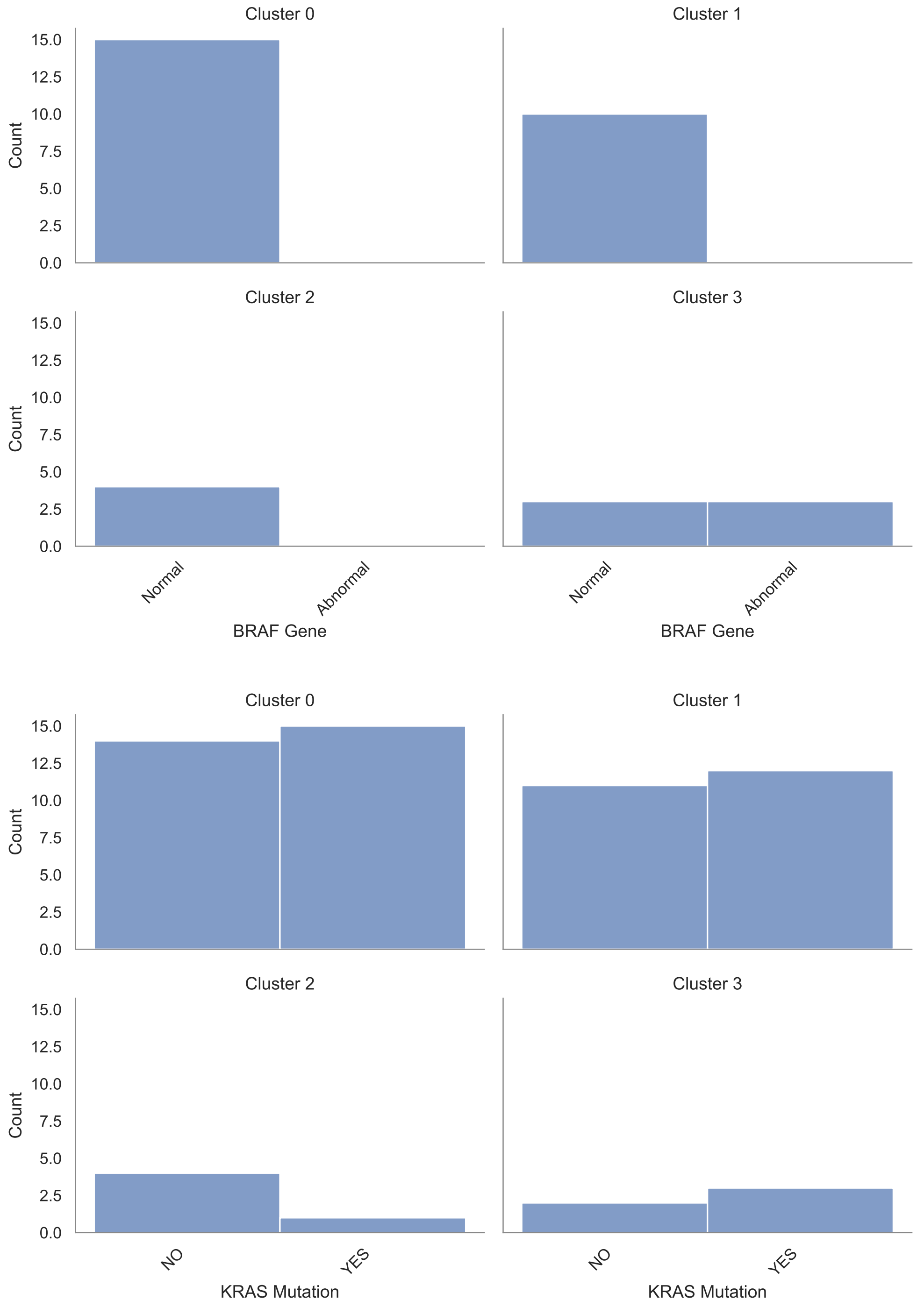

Fig. S11: **Distribution of BRAF and KRAS mutations in clusters generated by multi-omics data.** The all-surviving cluster 3 exhibits BRAF gene abnormality more frequently than other clusters. KRAS mutation status has no effect on the clusters.

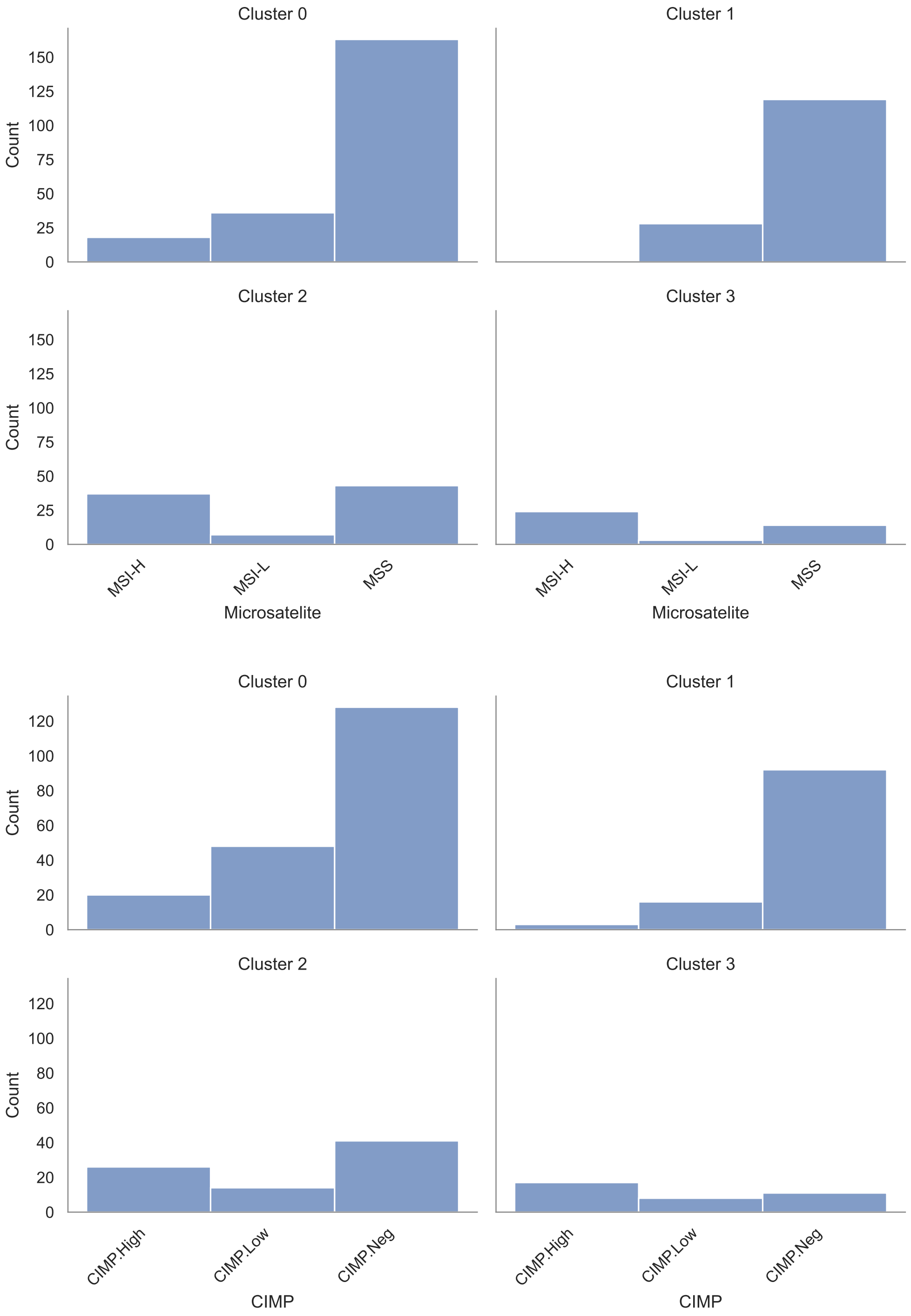

Fig. S12: **Distribution of MSI and CIMP in clusters generated by multi-omics data.** MSI and CIMP status have a significant effect on the clusters, with both the all-surviving cluster 3 and poor 10-year survival cluster 2 showing high MSI and high CIMP.

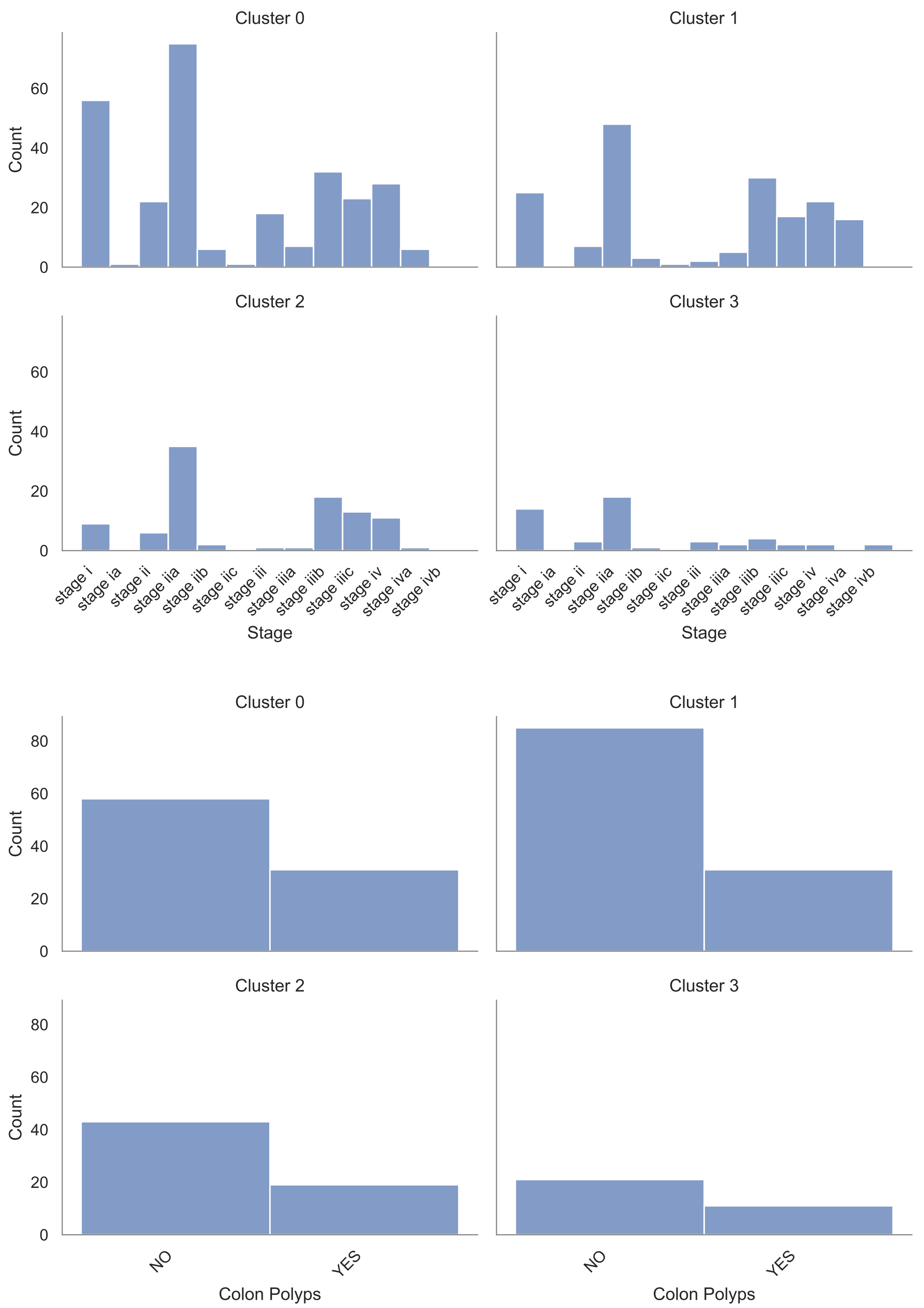

Fig. S13: **Distribution of stage and presence of colon polyps in clusters generated by multi-omics data.** Tumour stage is distinctly distributed, with the all-surviving cluster 3 showing majority early stage tumours. The presence of colon polyps have no significant effect on the clusters.

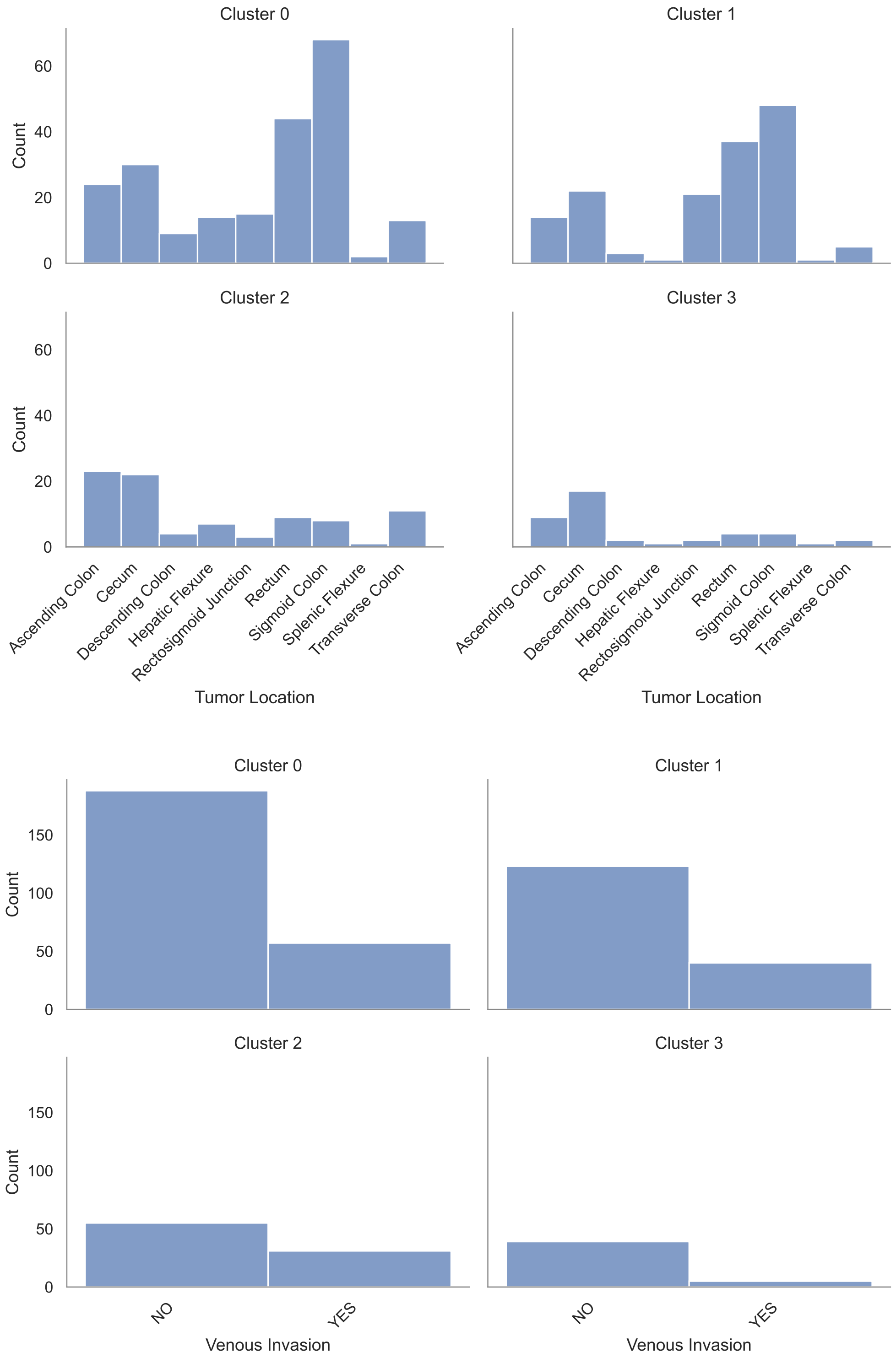

Fig. S14: **Distribution of tumour location and venous invasion in clusters generated by multi-omics data.** Both tumour location and venous invasion of tumours have a significant effect on the clusters. The all-surviving cluster 3 tumours are primarily located in ascending colon and cecum areas and patients of this cluster tend to show no venous invasion.

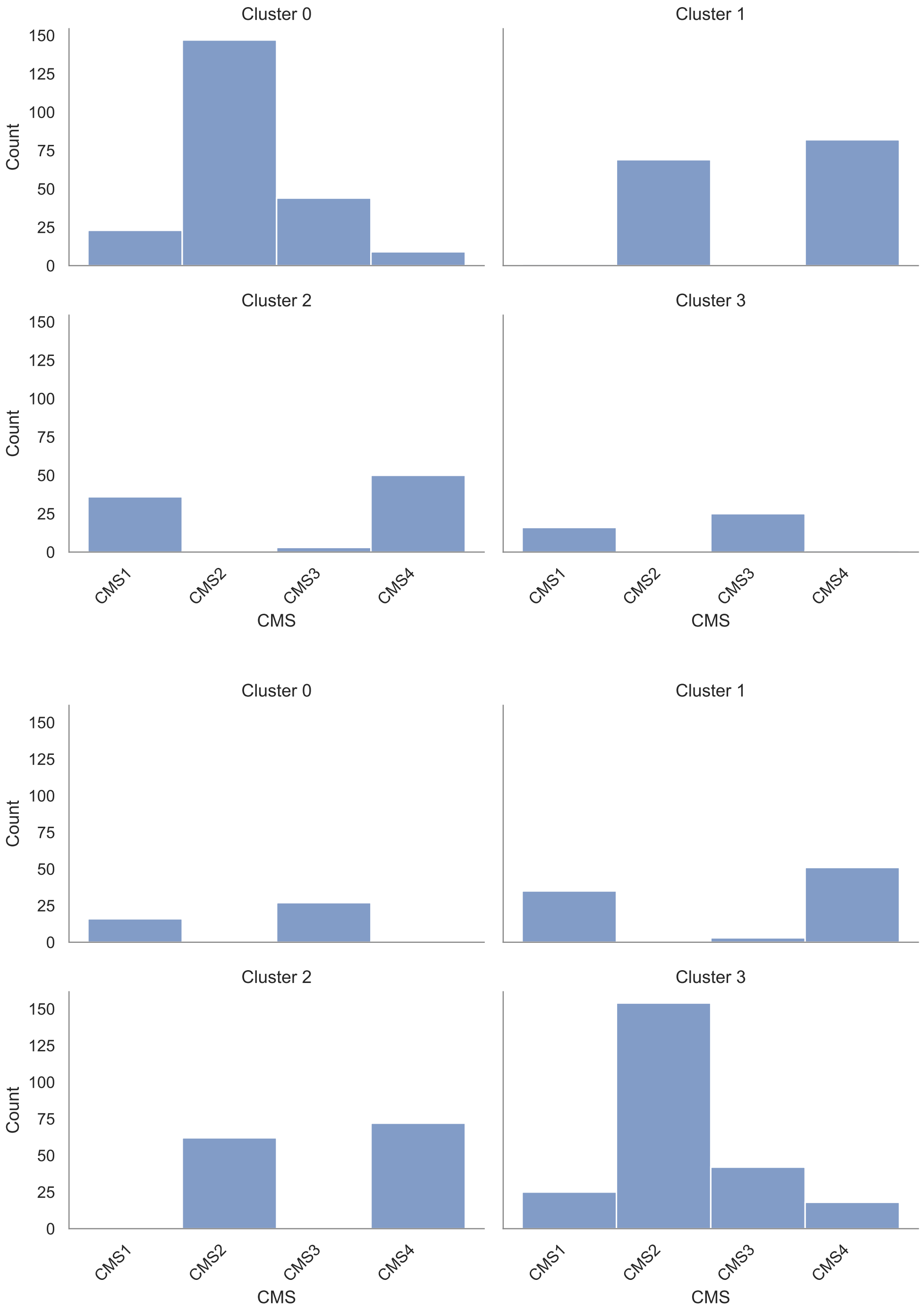

Fig. S15: **Distribution of CMS in clusters generated by multi-omics and multi-modal data.** CMS2 is visibly absent from the all-surviving clusters (3 for multi-omics and 0 for multi-modal) and we see the MSI-high CMS1 distributed between these two clusters.

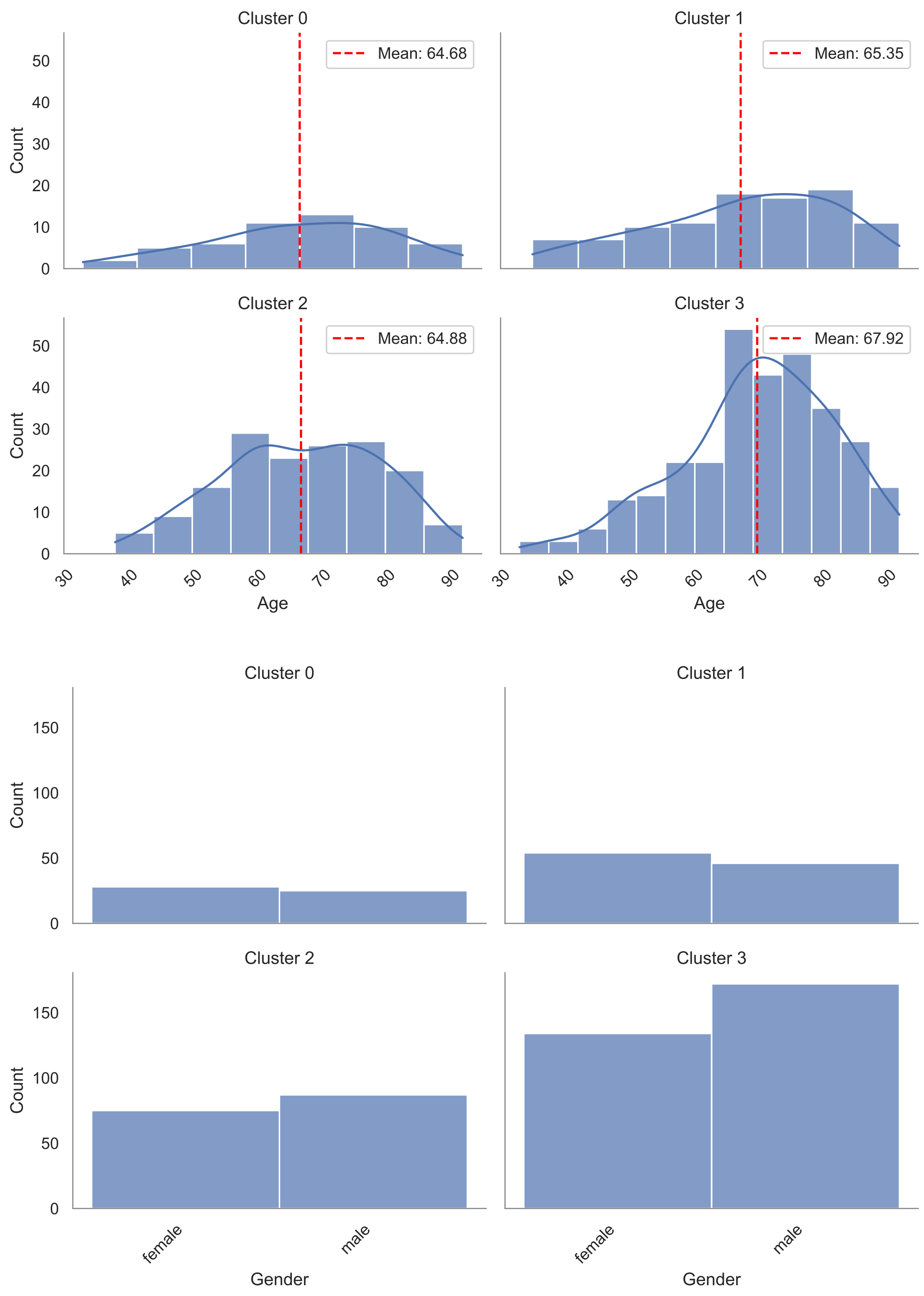

Fig. S16: **Distribution of age and gender in clusters generated by multi-modal data.** There is no bias towards key confounding factors such as age and gender on disease-specific survival in the all-surviving cluster identified by multi-modal data.

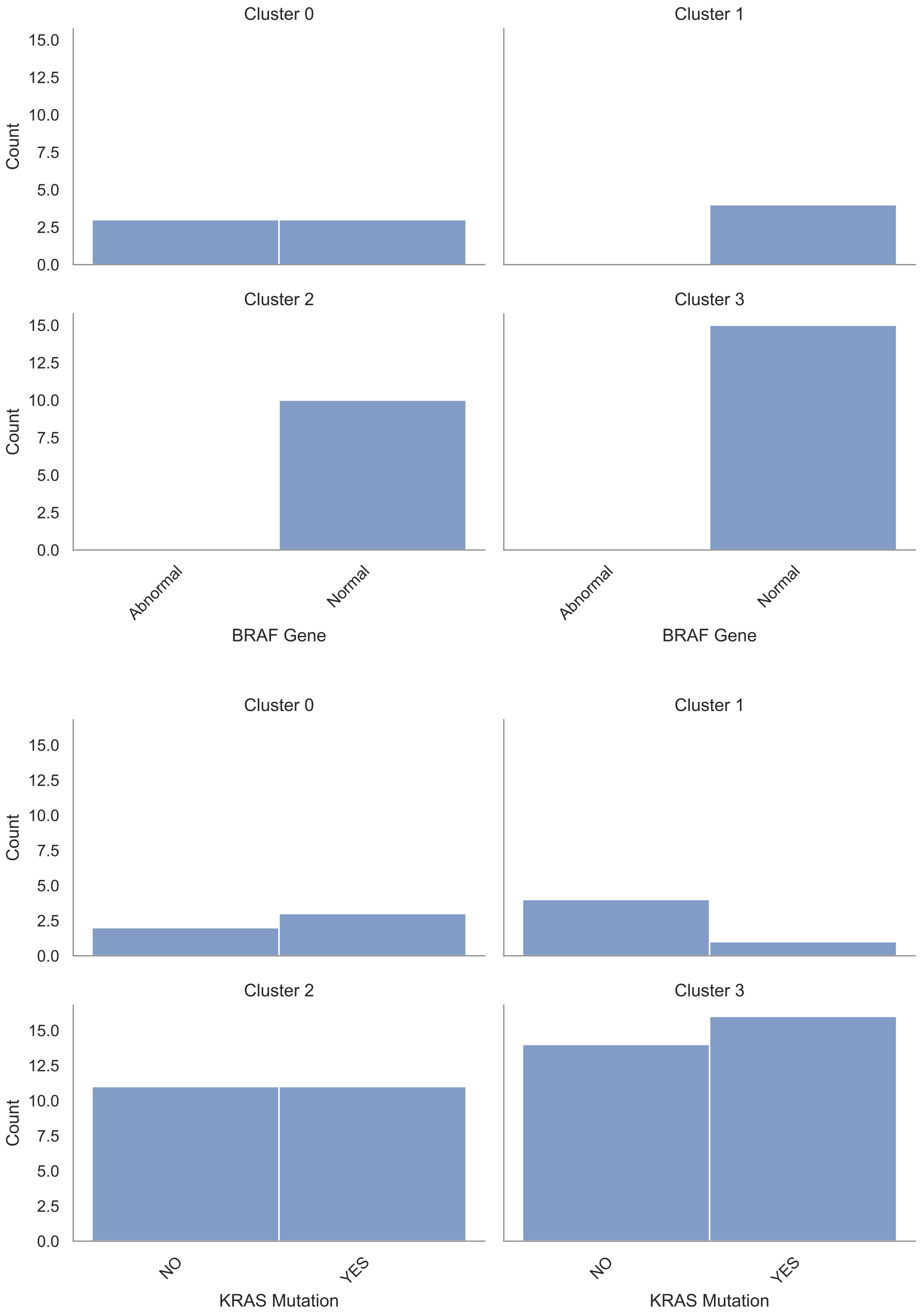

Fig. S17: **Distribution of BRAF and KRAS mutations in clusters generated by multi-modal data.** The all-surviving cluster 0 exhibits BRAF gene abnormality more frequently than other clusters. KRAS mutation status has no effect on the clusters.

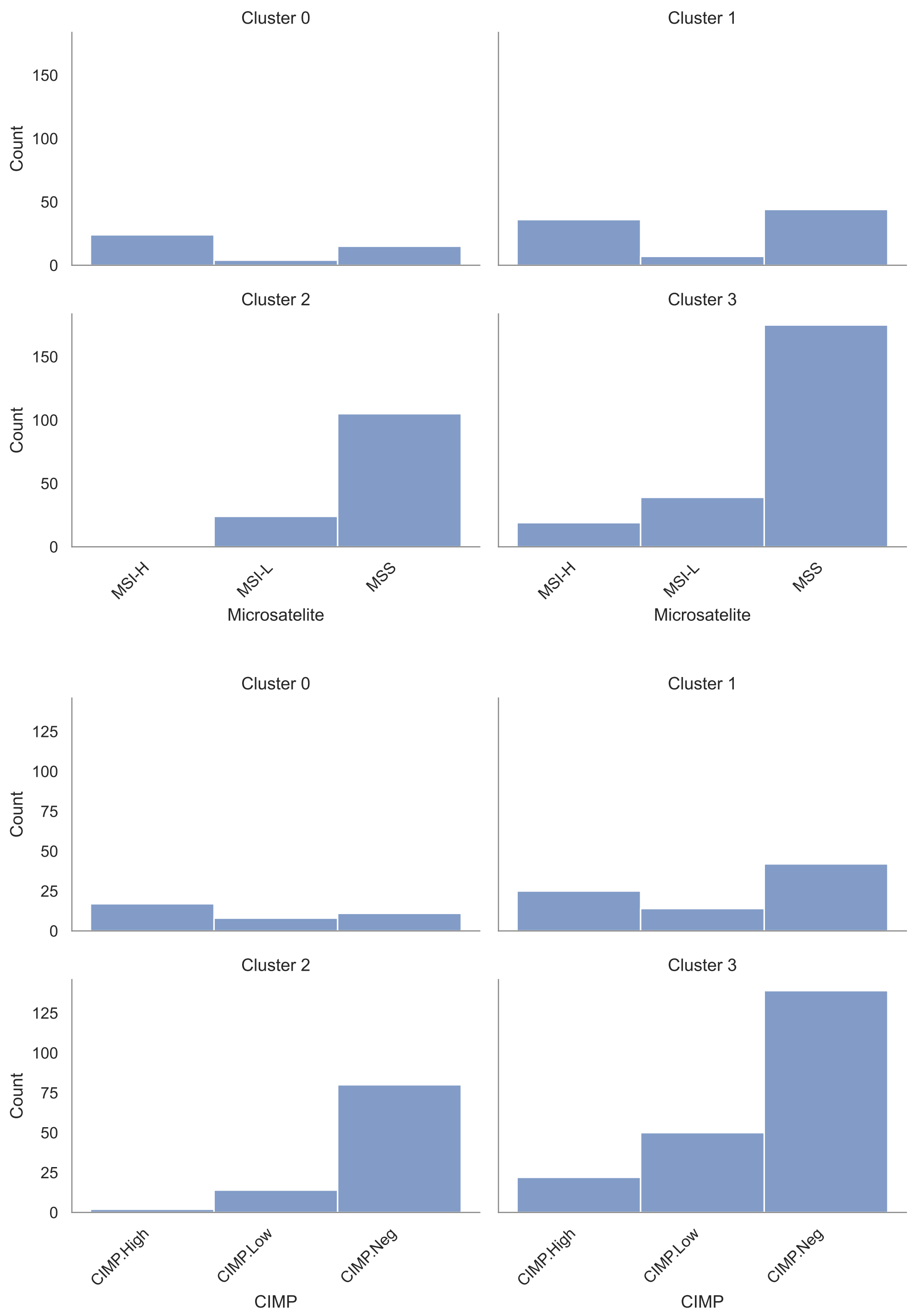

Fig. S18: **Distribution of MSI and CIMP in clusters generated by multi-modal data.** MSI and CIMP status have a significant effect, with both the all-surviving cluster 0 and poor 10-year survival cluster 1 showing high MSI and high CIMP.

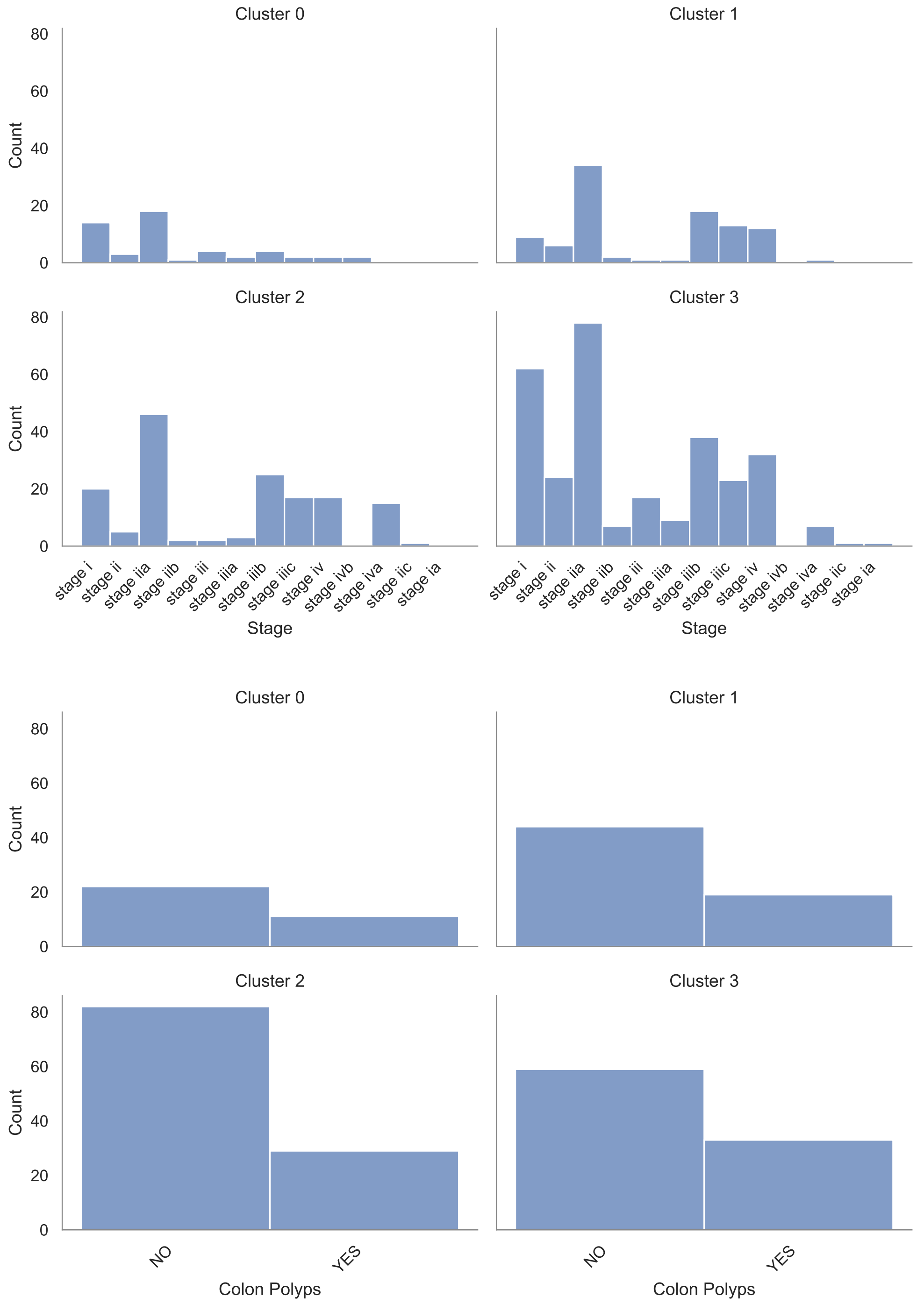

Fig. S19: **Distribution of stage and presence of colon polyps in clusters generated by multi-modal data.** Tumour stage is distinctly distributed, with the all-surviving cluster 0 showing majority early stage tumours. The presence of colon polyps have no significant effect on the clusters.

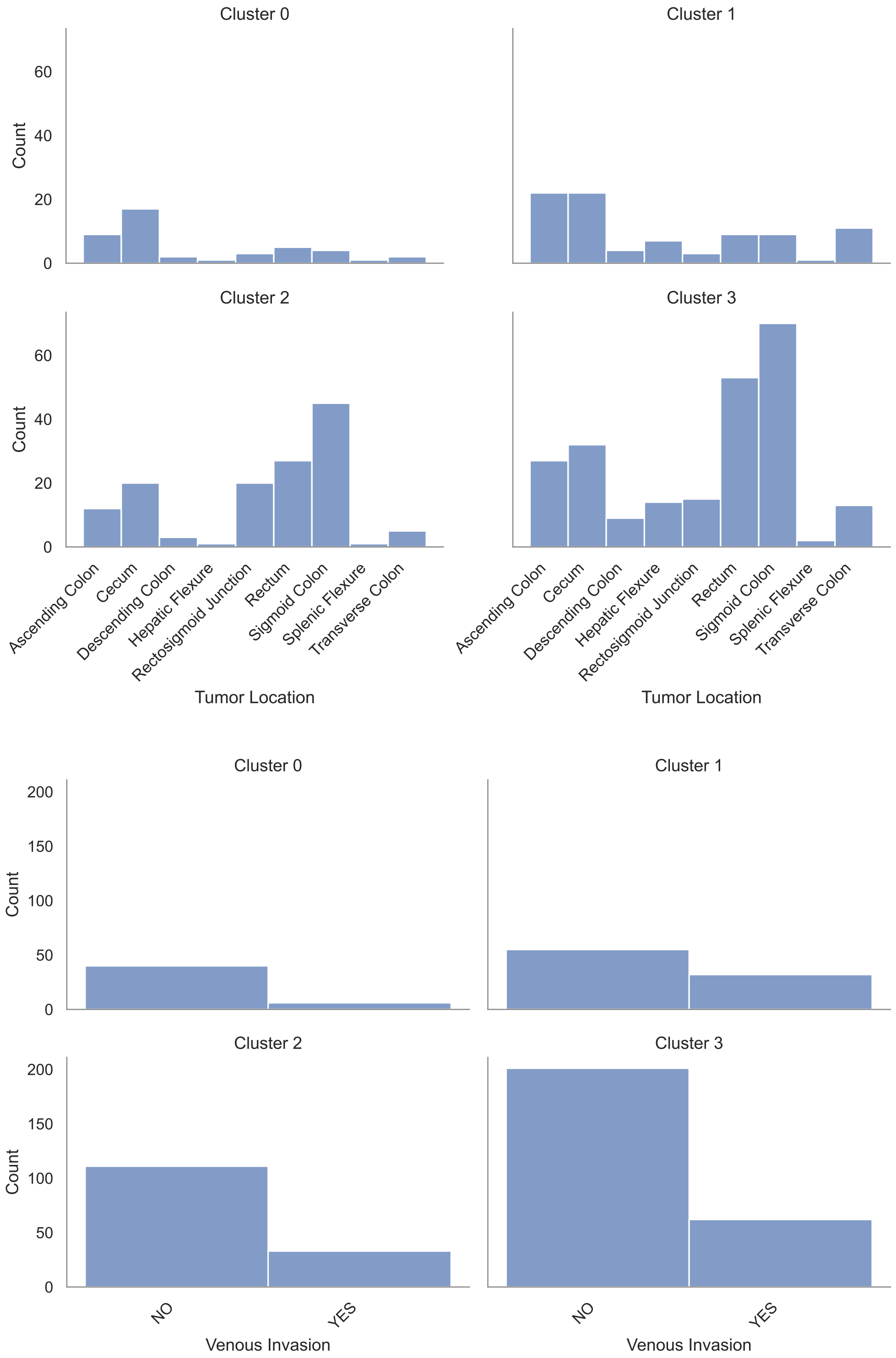

Fig. S20: **Distribution of tumour location and venous invasion in clusters generated by multi-modal data.** Both tumour location and venous invasion of tumours have a significant effect on the clusters. The all-surviving cluster 0 tumours are primarily located in ascending colon and cecum areas and patients of this cluster tend to show no venous invasion.

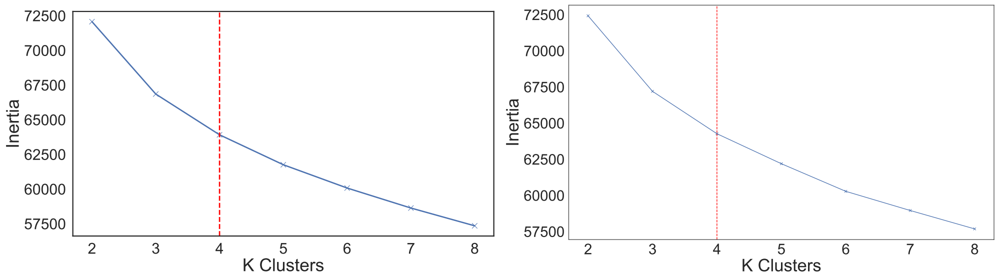

a) Normalised count multi-modal dataset. b) Normalised TF-IDF multi-modal dataset.

Fig. S21: **Optimal number of clusters $K = 4$.** For each dataset, the optimal number of clusters is found using the elbow method by plotting the sum of squared errors (inertia) against the number of clusters.

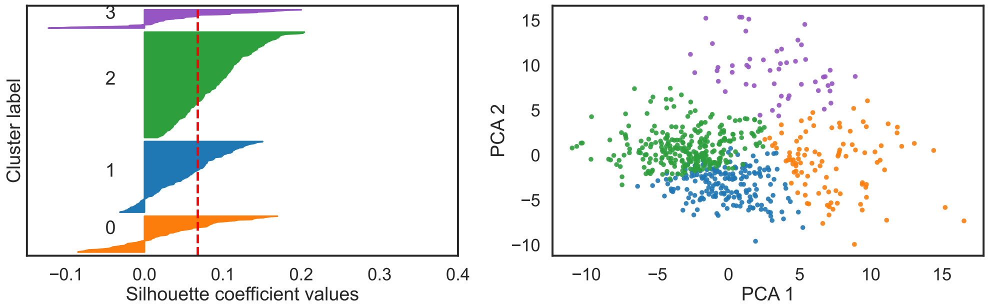

a) Normalised count multi-modal dataset.

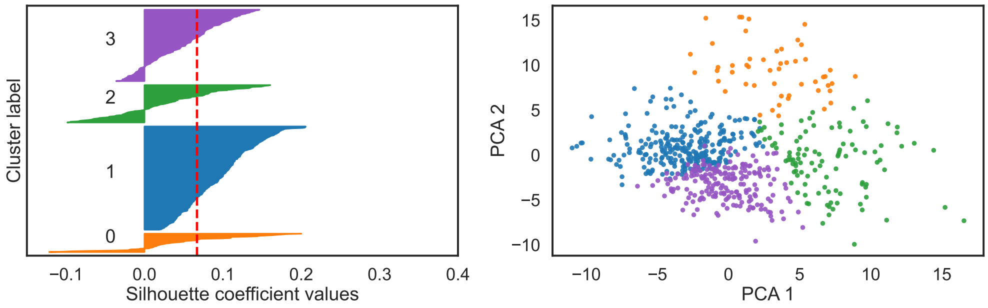

b) Normalised TF-IDF multi-modal dataset.

Fig. S22: **PCA visualisation of the optimal number of clusters for each dataset.** For each dataset, clustering is performed with the optimal number of clusters and visualised with principal component analysis. Additionally, a silhouette analysis is carried out to measure the tightness of the clusters.

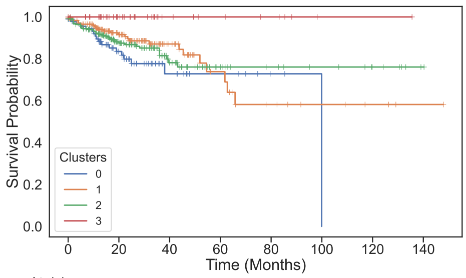

At risk								
Cluster 0	92	47	15	8	2	0	0	0
Cluster 1	179	102	38	15	8	5	3	1
Cluster 2	260	139	43	19	13	10	6	1
Cluster 3	47	28	10	7	5	1	1	0

a) Normalised count multi-modal dataset.

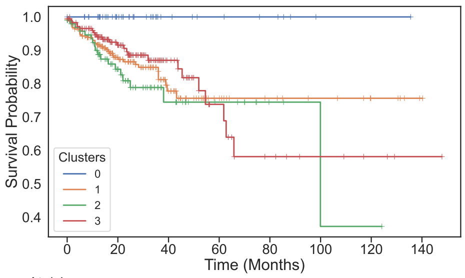

At risk								
Cluster 0	47	28	10	7	5	1	1	0
Cluster 1	255	136	42	18	12	9	5	1
Cluster 2	96	51	17	9	3	1	1	0
Cluster 3	180	101	37	15	8	5	3	1

b) Normalised TF-IDF multi-modal dataset.

Fig. S23: **Cluster-survival association study.** Kaplan-Meier plots of disease-specific survival (DSS) across clusters found by bag-of-patch image-based multi-modal datasets. The datasets are able to identify an all-surviving cluster of patients with high significance.

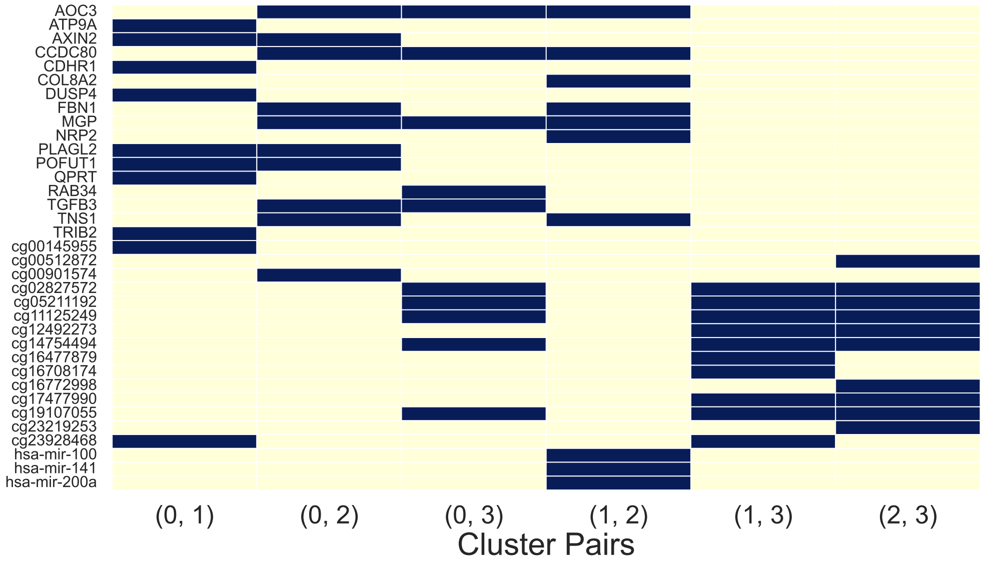

a) Normalised count multi-modal dataset.

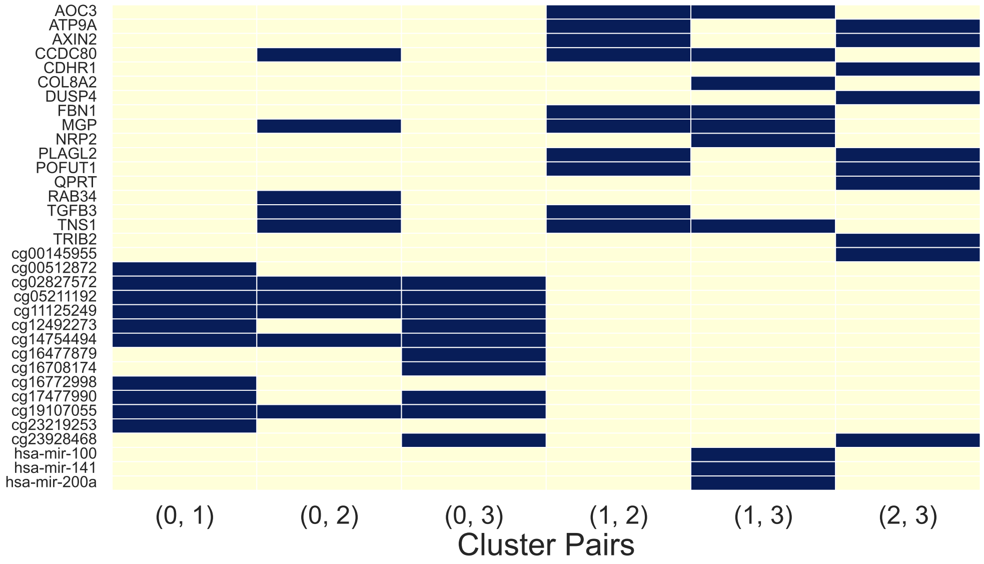

b) Normalised TF-IDF multi-modal dataset.

Fig. S24: Feature importance binary heatmap for multi-modal datasets with bag-of-patches image representation. The union of the top 10 features for each cluster pair is visualised against the cluster pair to which it contributes to qualitatively asses degree of overlap.

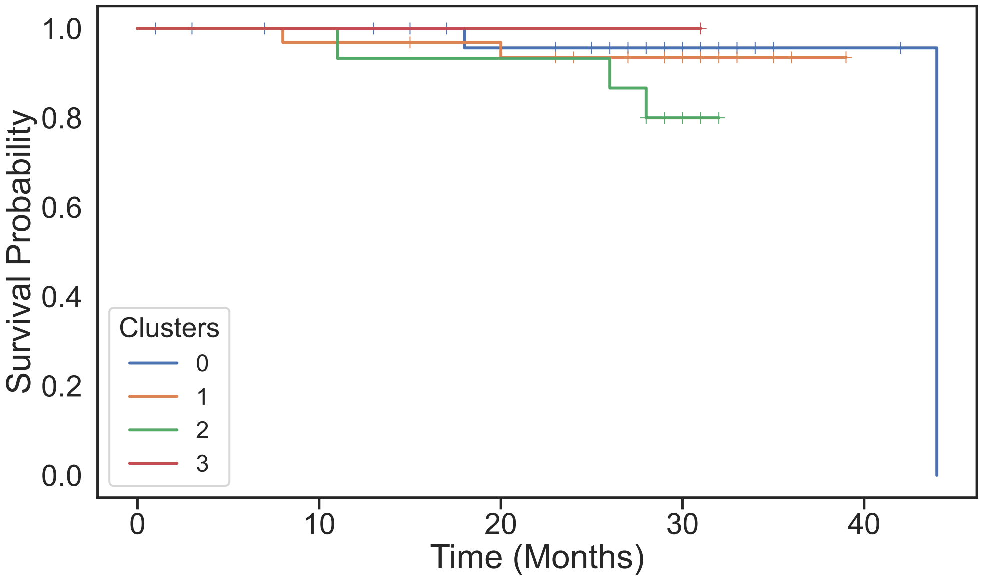

At risk					
Cluster 0	52	49	43	19	2
Cluster 1	32	31	28	20	0
Cluster 2	15	15	14	7	0
Cluster 3	1	1	1	1	0

Fig. S25: Kaplan-Meier curves for CPTAC data. Cluster membership is derived using a K-Nearest Neighbours classifier trained on TCGA data. We see similar trends as with TCGA data - an all-surviving cluster (albeit only with 1 patient) and a cluster having survival probability 0 after approximately 45 months.

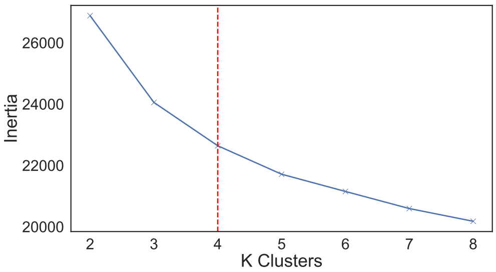

a) DNA methylation.

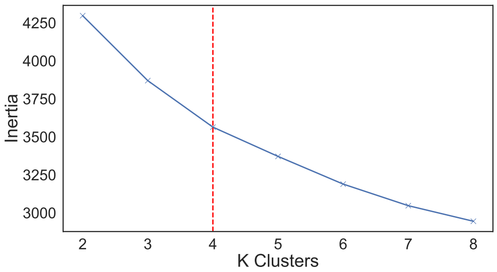

b) Protein expression.

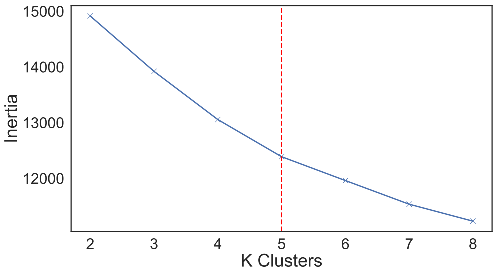

c) miRNA expression.

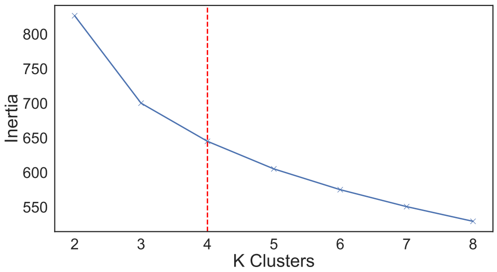

d) Whole slide images.

Fig. S26: **Finding optimal number of clusters.** For each dataset, the optimal number of clusters is found using the elbow method by plotting the sum of squared errors (inertia) against the number of clusters. $K = 4$ for (a), (b) and (d), and $K = 5$ for (c).

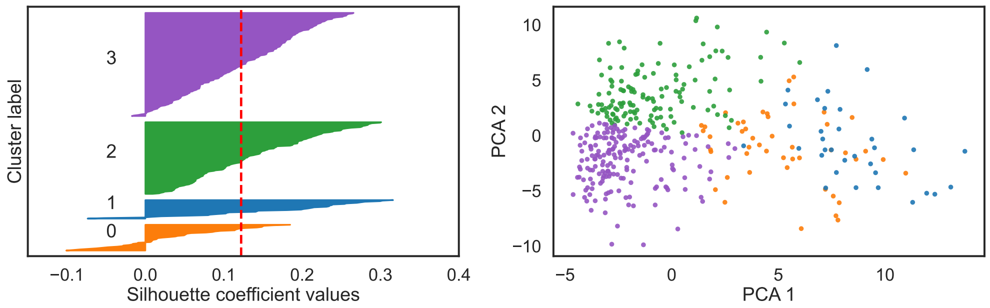

a) DNA methylation.

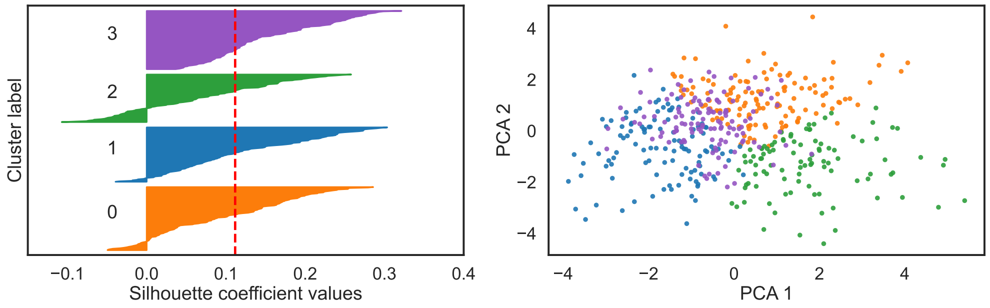

b) Protein expression.

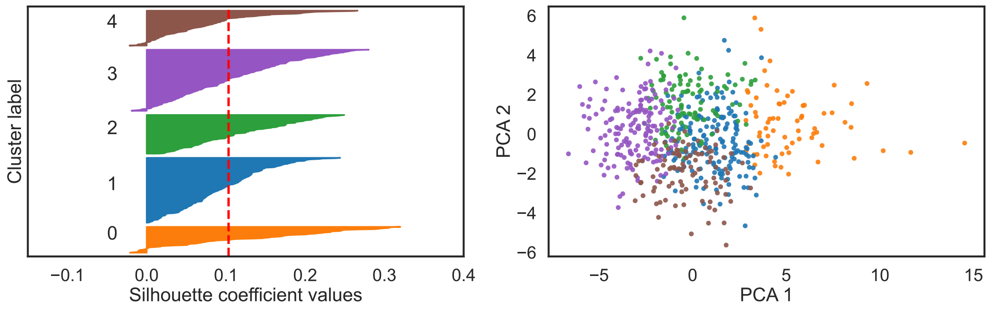

c) miRNA expression.

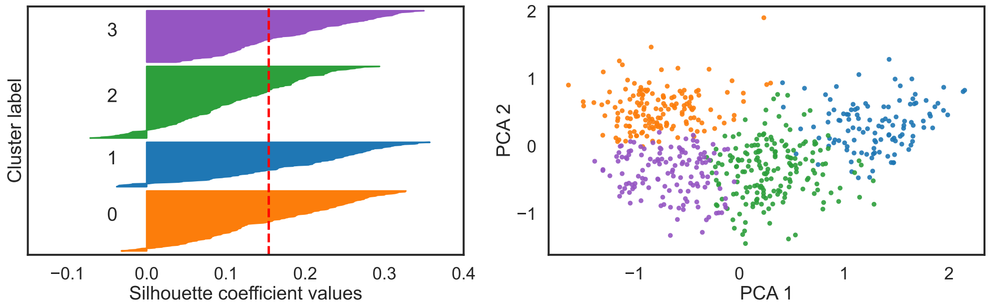

d) Whole slide images.

Fig. S27: **PCA visualisation of the optimal number of clusters for each dataset.** For each dataset, clustering is performed with the optimal number of clusters and visualised with principal component analysis. Additionally, a silhouette analysis is carried out to measure the tightness of the clusters.

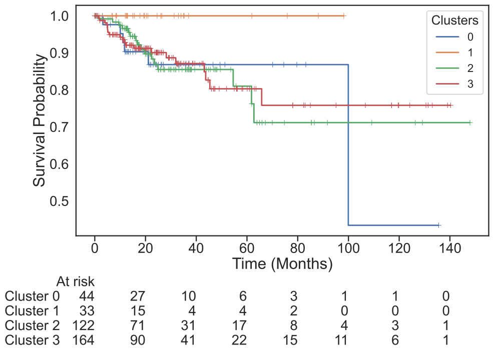

a) DNA methylation.

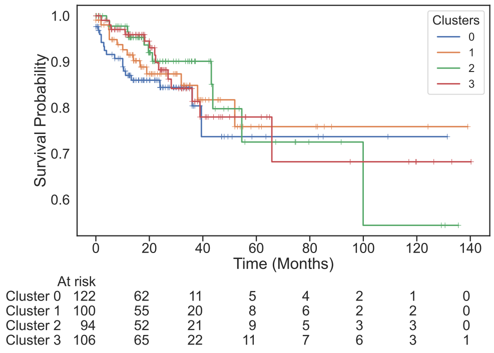

b) Protein expression.

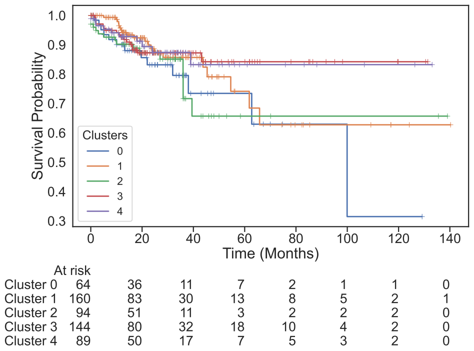

c) miRNA Expression.

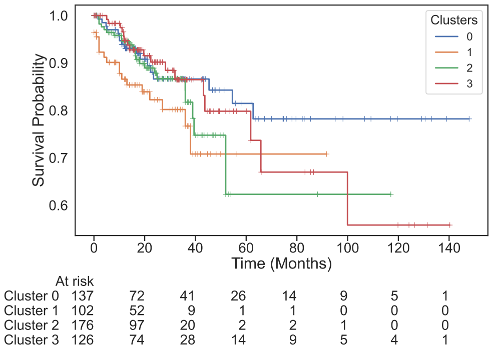

d) Whole slide images.

Fig. S28: **Cluster-survival association study.** Kaplan-Meier plots of disease-specific survival (DSS) across clusters found by different unimodal datasets. The DNA methylation dataset is able to identify a cluster of patients who do not experience any event.

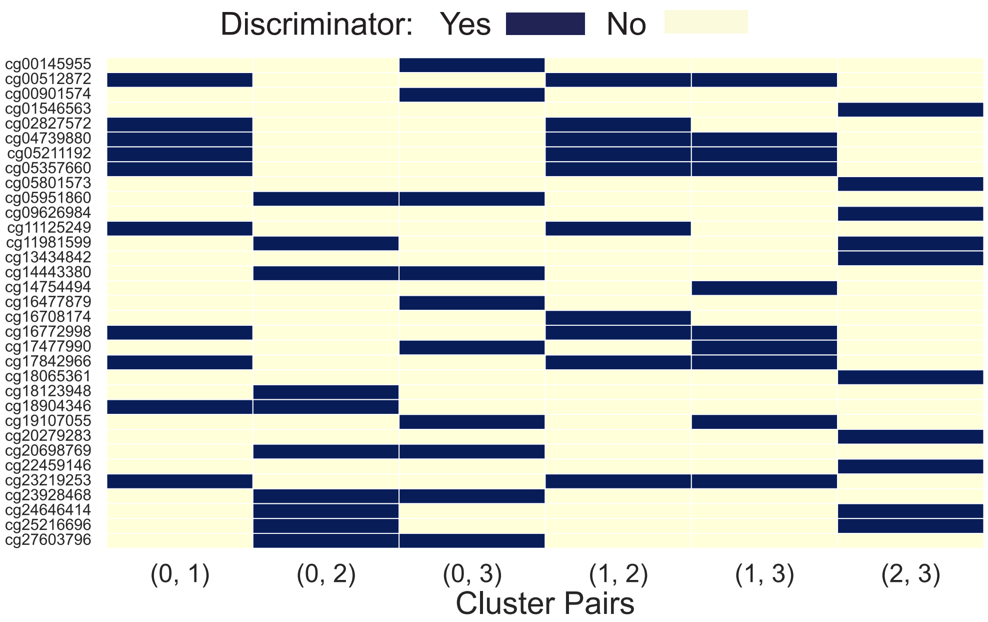

Fig. S29: Feature importance binary heatmap for unimodal DNA methylation dataset.

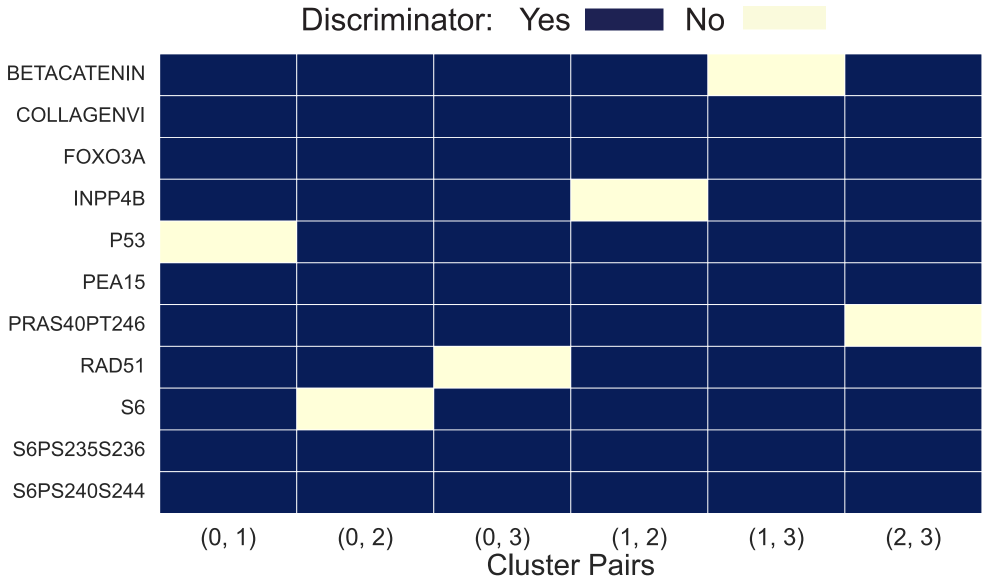

Fig. S30: Feature importance binary heatmap for unimodal protein expression dataset.

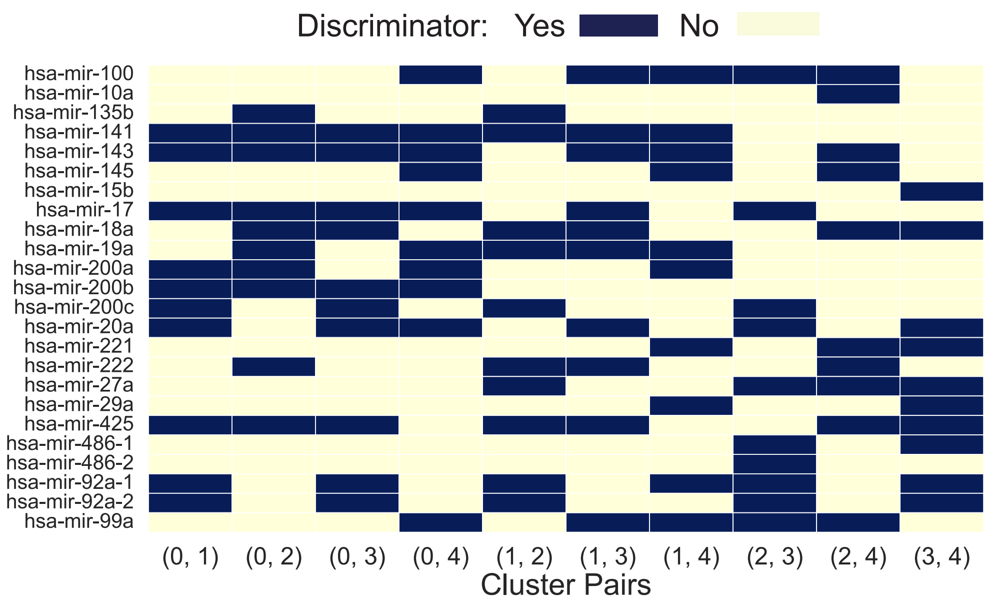

Fig. S31: Feature importance binary heatmap for unimodal miRNA expression dataset. The union of the top 10 features for each cluster pair is visualised against the cluster pair to which it contributes to qualitatively asses degree of overlap.

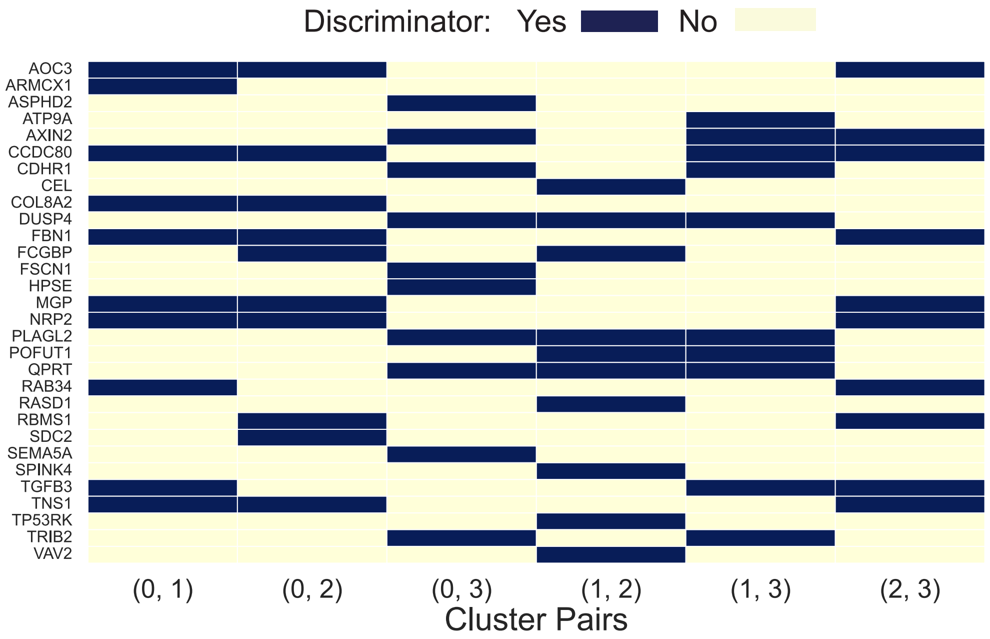

Fig. S32: Feature importance binary heatmap for unimodal gene expression dataset. The union of the top 10 features for each cluster pair is visualised against the cluster pair to which it contributes to qualitatively asses degree of overlap.

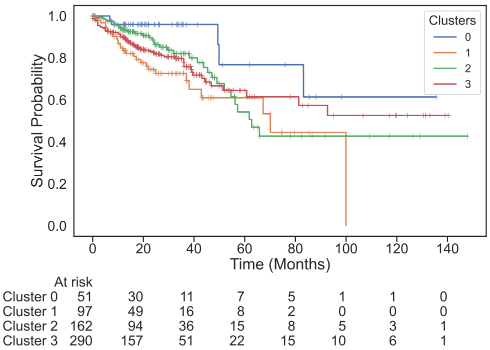

a) Multi-modal dataset.

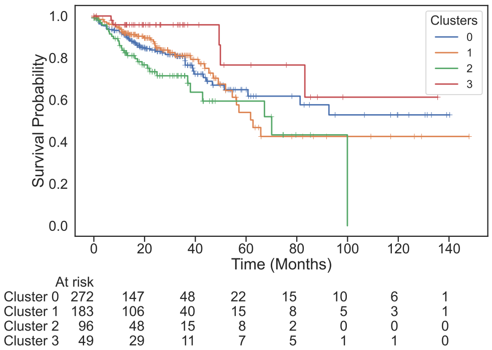

b) Multi-omics dataset.

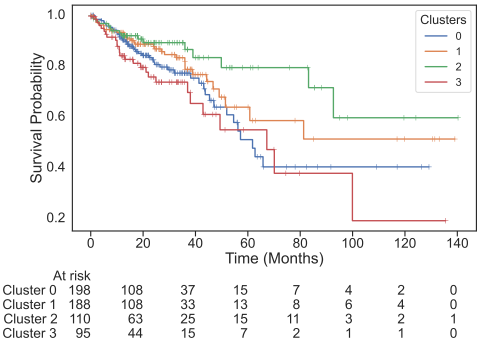

c) Gene expression with 40 gene markers.

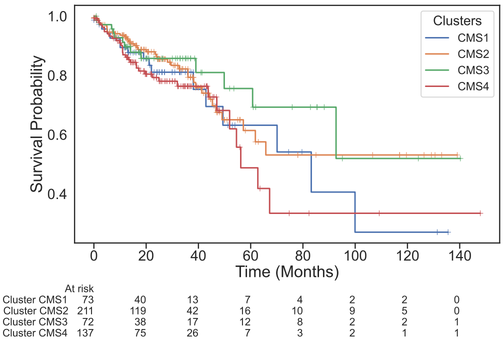

d) Consensus molecular subtypes.

Fig. S33: **Cluster-survival association study.** Kaplan-Meier plots of overall survival (OS) across clusters found by different unimodal datasets.

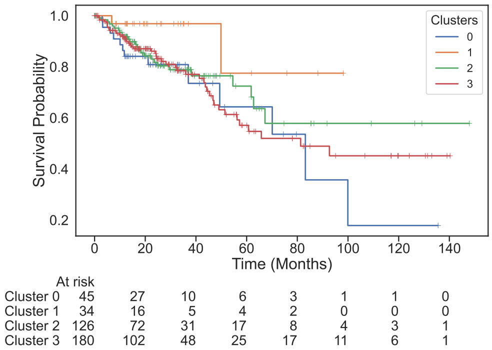

a) DNA methylation.

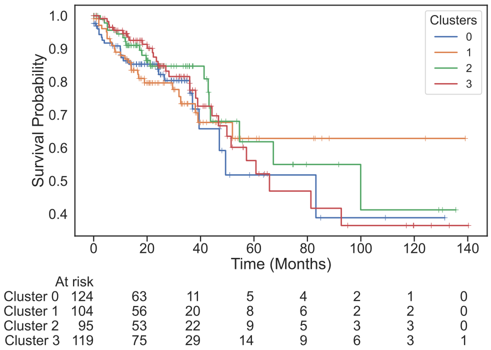

b) Protein expression.

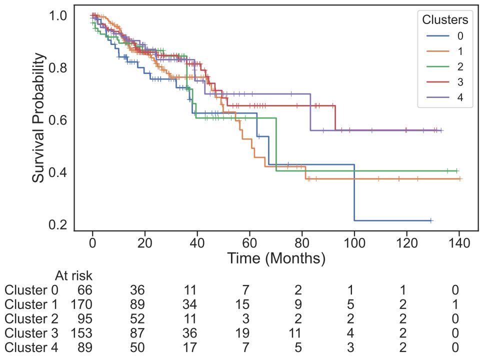

c) miRNA Expression.

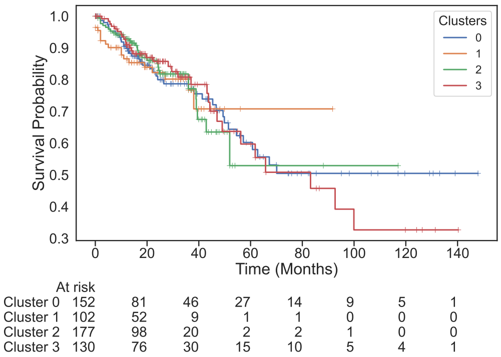

d) Whole slide images.

Fig. S34: **Cluster-survival association study.** Kaplan-Meier plots of overall survival (OS) across clusters found by different unimodal datasets.

References

[1] Buechler, S. A. *et al.* Colotype: a forty gene signature for consensus molecular subtyping of colorectal cancer tumors using whole-genome assay or targeted rna-sequencing. *Scientific reports* **10**, 1–13 (2020).

[2] Zhang, B. *et al.* Proteogenomic characterization of human colon and rectal cancer. *Nature* **513**, 382–387 (2014).

[3] Li, X. *et al.* A modified protein marker panel to identify four consensus molecular subtypes in colorectal cancer using immunohistochemistry. *Pathology-Research and Practice* **220**, 153379 (2021).

[4] Clarke, C. N. *et al.* Proteomic features of colorectal cancer identify tumor subtypes independent of oncogenic mutations and independently predict relapse-free survival. *Annals of surgical oncology* **24**, 4051–4058 (2017).

[5] Wang, X. *et al.* Identification of the mirna signature and key genes in colorectal cancer lymph node metastasis. *Cancer Cell International* **21**, 1–12 (2021).

[6] Liang, J. *et al.* Epigenetically regulated mir-1247 functions as a novel tumour suppressor via mycbp2 in methylator colon cancers. *British journal of cancer* **119**, 1267–1277 (2018).

[7] Gasparello, J. *et al.* A distinctive microrna (mirna) signature in the blood of colorectal cancer (crc) patients at surgery. *Cancers* **12**, 2410 (2020).

[8] Luo, X., Burwinkel, B., Tao, S. & Brenner, H. Microrna signatures: novel biomarker for colorectal cancer? *Cancer epidemiology, biomarkers & prevention* **20**, 1272–1286 (2011).

[9] Sheng, S. *et al.* Mir-144 inhibits growth and metastasis in colon cancer by down-regulating smad4. *Bioscience Reports* **39**, BSR20181895 (2019).

[10] Cui, H. *et al.* Igf2-derived mir-483 mediated oncofunction by suppressing dlc-1 and associated with colorectal cancer. *Oncotarget* **7**, 48456 (2016).

[11] Huang, X. *et al.* Dissecting mirna signature in colorectal cancer progression and metastasis. *Cancer letters* **501**, 66–82 (2021).

[12] Hajebi Khaniki, S., Shokoohi, F., Esmaily, H. & Kerachian, M. A. Analyzing aberrant dna methylation in colorectal cancer uncovered intangible heterogeneity of gene effects in the survival time of patients. *Scientific Reports* **13**, 22104 (2023).

[13] Ma, Y. *et al.* Genome wide identification of novel dna methylation driven prognostic markers in colorectal cancer. *Scientific Reports* **14**, 15654 (2024).

[14] Baharudin, R. *et al.* Epigenome-wide dna methylation profiling in colorectal cancer and normal adjacent colon using infinium human methylation 450k. *Diagnostics* **12**, 198 (2022).

[15] Onwuka, J. U. *et al.* A panel of dna methylation signature from peripheral blood may predict colorectal cancer susceptibility. *BMC cancer* **20**, 1–11 (2020).

[16] van Den Berg, I. *et al.* A panel of dna methylation markers for the classification of consensus molecular subtypes 2 and 3 in patients with colorectal cancer. *Molecular oncology* **15**, 3348–3362 (2021).

[17] Oliveira, C. *et al.* Kras and braf oncogenic mutations in mss colorectal carcinoma progression. *Oncogene* **26**, 158–163 (2007).

[18] Smeby, J. *et al.* Cms-dependent prognostic impact of kras and brafv600e mutations in primary colorectal cancer. *Annals of Oncology* **29**, 1227–1234 (2018).

[19] Drescher, K. M., Sharma, P. & Lynch, H. T. Current hypotheses on how microsatellite instability leads to enhanced survival of lynch syndrome patients. *Journal of Immunology Research* **2010**, 170432 (2010).

[20] Popat, S., Hubner, R. & Houlston, R. Systematic review of microsatellite instability and colorectal cancer prognosis. *Journal of clinical oncology* **23**, 609–618 (2005).

[21] Rhyu, M. S. Molecular mechanisms underlying hereditary nonpolyposis colorectal carcinoma. *JNCI: Journal of the National Cancer Institute* **88**, 240–251 (1996).

[22] Guinney, J. *et al.* The consensus molecular subtypes of colorectal cancer. *Nature medicine* **21**, 1350–1356 (2015).

[23] Csurka, G., Dance, C., Fan, L., Willamowski, J. & Bray, C. *Visual categorization with bags of keypoints*, 1–22 (Springer, Prague, Czech Republic, 2004).

[24] Sivic, J. & Zisserman, A. *Video google: A text retrieval approach to object matching in videos*, 1470–1477 (IEEE, 2003).

[25] Bouslimi, R., Messaoudi, A. & Akaichi, J. Using a bag of words for automatic medical image annotation with a latent semantic. *International Journal of Artificial Intelligence & Applications* **4**, 51 (2013).

[26] Pedregosa, F. *et al.* Scikit-learn: Machine learning in python. *the Journal of machine Learning research* **12**, 2825–2830 (2011).

[27] Satopaa, V., Albrecht, J., Irwin, D. & Raghavan, B. *Finding a "kneedle" in a haystack: Detecting knee points in system behavior*, 166–171 (IEEE, 2011).

[28] Edwards, N. J. *et al.* The cptac data portal: a resource for cancer proteomics research. *Journal of proteome research* **14**, 2707–2713 (2015).
